# Genotypic and Phenotypic Applications for the Differentiation and Species-Level Identification of *Achromobacter* for Clinical Diagnoses

**DOI:** 10.1371/journal.pone.0114356

**Published:** 2014-12-04

**Authors:** Margarita Gomila, Claudia Prince-Manzano, Liselott Svensson-Stadler, Antonio Busquets, Marcel Erhard, Deny L. Martínez, Jorge Lalucat, Edward R. B. Moore

**Affiliations:** 1 Microbiology, Department of Biology, University of the Balearic Islands, Palma de Mallorca, Islas Baleares, Spain; 2 Department of Infectious Diseases, Culture Collection University of Gothenburg (CCUG), Sahlgrenska Academy of the University of Gothenburg, Gothenburg, Sweden; 3 RIPAC-LABOR GmbH, Potsdam-Golm, Germany; 4 Mediterranean Institute for Advanced Studies (IMEDEA) (CSIC-UIB), Palma de Mallorca, Islas Baleares, Spain; University of Strathclyde, United Kingdom

## Abstract

The *Achromobacter* is a genus in the family *Alcaligenaceae*, comprising fifteen species isolated from different sources, including clinical samples. The ability to detect and correctly identify *Achromobacter* species, particularly *A. xylosoxidans,* and differentiate them from other phenotypically similar and genotypically related Gram-negative, aerobic, non-fermenting species is important for patients with cystic fibrosis (CF), as well as for nosocomial and other opportunistic infections. Traditional phenotypic profile-based analyses have been demonstrated to be inadequate for reliable identifications of isolates of *Achromobacter* species and genotypic-based assays, relying upon comparative 16S rRNA gene sequence analyses are not able to insure definitive identifications of *Achromobacter* species, due to the inherently conserved nature of the gene. The uses of alternative methodologies to enable high-resolution differentiation between the species in the genus are needed. A comparative multi-locus sequence analysis (MLSA) of four selected ‘house-keeping’ genes (*atpD*, *gyrB*, *recA*, and *rpoB*) assessed the individual gene sequences for their potential in developing a reliable, rapid and cost-effective diagnostic protocol for *Achromobacter* species identifications. The analysis of the type strains of the species of the genus and 46 strains of *Achromobacter* species showed congruence between the cluster analyses derived from the individual genes. The MLSA gene sequences exhibited different levels of resolution in delineating the validly published *Achromobacter* species and elucidated strains that represent new genotypes and probable new species of the genus. Our results also suggested that the recently described *A. spritinus* is a later heterotypic synonym of *A. marplatensis.* Strains were analyzed, using whole-cell Matrix-Assisted Laser Desorption/Ionization Time-Of-Flight mass spectrometry (MALDI-TOF MS), as an alternative phenotypic profile-based method with the potential to support the identifications determined by the genotypic DNA sequence-based MLSA. The MALDI-TOF MS data showed good accordance in strain groupings and identifications by the MLSA data.

## Introduction

The Gram-negative, aerobic, non-fermenting bacteria are ubiquitously present in various ecosystems, important for environmental and biotechnological applications and many of these microorganisms have become problematic in hospital settings. Species of *Pseudomonas*, *Burkholderia, Acinetobacter* and *Stenotrophomonas* are the leading nosocomial pathogens in this expanding group [1], [2] and genera of the family *Alcaligenaceae*, *i.e., Alcaligenes, Ralstonia, Achromobacter*, etc., are emerging, as well, as significant pathogens in notable patient populations [3], particularly those suffering from cystic fibrosis (CF). Recent studies have reported as many as 5 to 10% of colonizing bacteria in respiratory tract samples from CF patients are *Achromobacter* species [1]; earlier studies also reported approximately 5% of CF patients examined were colonized with *A. xylosoxidans* [4]; 3 to 4% of CF patients exhibit chronic colonizations and approximately 2% sporadic colonizations [5], [6]. Infections by *A. xylosoxidans* in CF patients have been observed to lead to decline in lung function [7], [8]. The ability to detect and correctly identify *Achromobacter* species, particularly *A. xylosoxidans,* and differentiate them from other phenotypically similar and genotypically related Gram-negative, aerobic, non-fermenting species is increasingly important. Misidentifications compromise infection control measures and confound efforts to recognise the epidemiology of infections. The growing number of species and increasing complexity of bacterial taxonomy and the expansion of virulence and antibiotic resistance present significant challenges, requiring new development and periodic optimisation of identification protocols for new, as well as already described taxa.

*Achromobacter* is one of 19 genera belonging to the family *Alcaligenaceae*, in the class *Betaproteobacteria* [9]–[12] and the taxonomy of *Achromobacter* has been closely intertwined with that of the genus *Alcaligenes* [10]; several species of *Alcaligenes* have been reclassified as *Achromobacter*. *Achromobacter* comprises 15 species: *A. xylosoxidans* (ex Yabuuchi and Ohyama 1971) Yabuuchi and Yano 1981, sp. nov., nom. rev. emend. (Type species of the genus) [10]; *A. ruhlandii* (Packer and Vishniac 1955) (Yabuuchi *et al*. 1998, comb. nov.) [10]; *A. piechaudii* (Kiredjian *et al.* 1986) *Yabuuchi et al.* 1998, comb. nov. [10]; *A. denitrificans* (Ruger and Tan 1983) Coenye *et al*. 2003, comb. nov. [13]; *A. insolitus* Coenye *et al*. 2003, sp. nov. [14]; *A. spanius* Coenye *et al.* 2003, sp. nov. [14]; *A. marplatensis* Gomila *et al*. 2011, sp. nov. [15]; *A. animicus*; *A. mucicolens*; *A. pulmonis*; and *A. spiritinus* Vandamme *et al.*, 2013a [16]. Recently, four new species have been described: *A. insuavis* sp. nov.; *A. aegrifaciens* sp. nov.; *A. anxifer* sp. nov.; and *A. dolens* Vandamme *et al.*, 2013b [17]. These species were isolated from different sources, including clinical samples. *A. xylosoxidans* is widely distributed in the environment and is also recognized to be an opportunistic human pathogen, associated with a variety of infections, including bacteraemia, meningitis, pneumonia, and peritonitis [18]–[20]. Nosocomial outbreaks attributed to disinfectant solutions, dialysis fluids, saline solutions and deionised water contaminated with this species have been described [21], [22]. *A. xylosoxidans* presents significant problems for persistent infection of the respiratory tract in persons with CF [4], although the precise role in contributing to pulmonary decline in this patient population is not clear. However, due to the well-known difficulties in differentiating the species of *Achromobacter*, it must be appreciated that isolates identified as ‘*A. xylosoxidans*’ may, in fact, comprise different *Achromobacter* species. *A. ruhlandii* is considered to be a soil inhabitant, although it has been associated also with human clinical conditions [23]. *A. piechaudii* has been isolated from human clinical samples, including blood, as well as from soil [24]. *A. insolitus* and *A. spanius* were isolated initially from leg wound and blood samples, respectively [14]. *A. denitrificans* strains are found typically in soil and water but can occasionally be found in human clinical samples [13], [23]. *A. animicus*, *A. mucicolens*, *A. pulmonis*, *A. spiritinus* [16], [25], *A. aegrifaciens*, *A. anxifer*, *A. dolens* and *A. insuavis* [17] were isolated from the sputa of patients with and without CF, as well as from water and sludge.

Traditional phenotypic-based analyses have been demonstrated to be inadequate for reliable, definitive identifications of *Achromobacter* species [12], [15], [25]. Recognizing the limitations of phenotypic-based identifications of bacteria, genotypic-based phylogeny has been recommended as the basis for the taxonomy of microorganisms [26]. While whole genome sequences ultimately will constitute the paramount reference standard for microbial phylogeny, sequence determinations of ‘biomarker’ genes, such as those for 16S rRNA, will continue to provide a basis for determining microbial phylogenetic relationships, taxonomy and identification. The comparative analyses of 16S rRNA gene sequences are used as part of the standard protocol by clinical laboratories, particularly for reliable, initial, estimates of the identifications of isolates. However, comparative 16S rRNA gene sequence analyses do not engender the resolution required to ensure definitive delineation of closely related bacterial species, due to the conserved nature of the gene; all known *Achromobacter* species exhibit sequence dissimilarities less than 1% to each other. Protein-coding ‘house-keeping’ gene sequence data provide higher-resolution differentiation for reliable identification of closely related species [27] and compilations of multiple gene sequences complement their differentiation [28]. During the course of this study, two research groups developed independent multiple-locus approaches to facilitate the analyses of *Achromobacter* species [25], [29]. While multi-locus sequence analysis (MLSA) and multi-locus sequence typing (MLST) approaches enhance the insight into the systematic relationships and population dynamics of bacterial taxa, such approaches are complex and not always practical for rapid and cost-effective microbial identifications for clinical diagnoses. The ideal DNA sequence-based protocol for clinical diagnostics would be one in which a single target gene would afford the resolution necessary for reliable species-level differentiation and identifications, elucidated by one or two sequencing reactions. The focus of this study was to assess selected house-keeping gene sequences to identify marker genes that can be used for reliable differentiation and identification of clinical isolates of *Achromobacter* species.

Analyses, using Matrix-Assisted Laser Desorption/Ionization Time-Of-Flight mass spectrometry (MALDI-TOF MS) have not been evaluated previously for the potential for identification of the individual species in the *Achromobacter* genus. The *Achromobacter* species reference data included in the VITEK MS IVD (BioMérieux, Inc.) identification system are limited to *A. xylosoxidans* and *A. denitrificans*, although the SARAMIS software (Anagnostec GmbH/bioMérieux, Inc.) [30] included the seven species of *Achromobacter* that were validly published until 2010; the MALDI Biotyper (Bruker Corp.) identification system includes *A. xylosoxidans*, *A. ruhlandii*, *A. piechaudii*, *A. denitrificans*, *A. insolitus*, and *A. spanius* [31]. A comprehensive assessment of MALDI-TOF MS for identifying all species of *Achromobacter* offers a complementary methodology for comparison with DNA sequence-based approaches. The MALDI-TOF MS identifications of the *Achromobacter* species, analysed in this study are compared and correlated with the MLSA identifications.

## Materials and Methods

### Bacterial strains and growth conditions

All strains used in this study were obtained from the Culture Collection University of Gothenburg (CCUG; www.ccug.se), including the type strains of eleven validly published species of the genus *Achromobacter*: *A. xylosoxidans* CCUG 56438^T^ (the type species of the genus); *A. ruhlandii* CCUG 57103^T^; *A. piechaudii* CCUG 724^T^; *A. denitrificans* CCUG 407^T^; *A. insolitus* CCUG 47057^T^; *A. spanius* CCUG 47062^T^; *A. marplatensis* CCUG 56371^T^; *A. animicus* CCUG 61966^T^; *A. mucicolens* CCUG 61961^T^; *A. pulmonis* CCUG 61972^T^; *A. spiritinus* CCUG 61968^T^; and 46 well-characterized strains of *Achromobacter* species of clinical and environmental origin. The sources of these isolates are diverse, including clinical samples (human sputum, respiratory tract from CF patients, synovial fluid samples, eye secretions, mucous samples of human cheek, choledochal cyst secretions, human tracheal secretions, pleural fluids, human wounds, bronchoalveolar lavage, otitis media, and environmental samples (soil, chicken tracheal samples, laboratory wash system) (Table S1). The type strain of the type species of the genus *Bordetella*, *B. pertussis* CCUG 30873^T^, was included as an out-group. Isolates were cultured on 5% Blood Agar and on Nutrient Agar media, at 30°C, 24–48 hours.

### DNA extraction, PCR amplification and DNA sequencing

Bacterial genomic DNA for PCR amplifications was extracted as previously described [32]. Five genes were selected for the MLSA: 16S rRNA; *atpD* (encoding the β subunit of ATP synthase), *gyrB* (encoding the β-subunit of DNA gyrase); *recA* (encoding the α-subunit of recombinase); and *rpoB* (encoding the β subunit of the RNA polymerase). Primers used for PCR amplifications and sequencing are listed in Table 1. PCR amplification primers for 16S rRNA genes, *gyrB* and *rpoB* were described previously [33]–[35]. Primers for the amplification of *gyrB* and *rpoB* were modified, according to the sequences of analyzed type strains. Primers used for amplification of *atpD* and *recA* were derived through alignment of the gene sequences from available whole genome sequence data of bacteria in the family *Alcaligenaceae*: *Bordetella avium* 197N; *Bordetella pertussis* Tohama I; *Bordetella petrii* DSM 12804; *Burkholderia cenocepacia* AU1054; *Burkholderia mallei* ATCC 23344; *Burkholderia ambifaria* MC40-6; *Ralstonia solanacearum* GMI1000; *Ralstonia pickettii* 12J; *Cupriavidus metallidurans* CH34; *Cupriavidus necator* H16; *Cupriavidus taiwanensis* LMG 19424; *Janthinobacterium* sp.; and *Herminiimonas arsenicoxydans* ULPAs1. Primers were designed for amplification of the sequence regions with the highest incidence of polymorphic sites in these genes. Internal sequencing primers were designed according to the alignments of the sequences of the type strains. PCR amplifications were carried out as previously described [15]. The amplification reactions were performed in an Eppendorf thermocycler, with an initial denaturation step of 5 min at 94°C, followed by 35 cycles of 1 min at 94°C, 1 min at the annealing temperature for each gene (55°C for 16S rRNA gene and 58°C for *atpD*, *gyrB* and *recA* genes) and 1 min 30 sec at 72°C. For the *atpD* gene, the initial denaturation step was 95°C for 2 min, followed by 35 cycles of 1 min at 95°C, 1 min at 60°C and 1 min 30 s at 72°C. Following the amplification cycles, samples were incubated at 72°C for 10 min and then cooled to 4°C. PCR amplicons of the targeted genes were purified, using the MultiScreen HTS PCR 96-well Filter Plates (Millipore) and sequenced directly, using the ABI PRISM BigDye Terminator Cycle Sequencing Kit version 3.1, according to the instructions of the manufacturer (Applied Biosystems, Inc.). Sequences were determined, using an ABI PRISM 3100 Avant-Genetic Analyzer and a 3130 Genetic Analyzer (Applied Biosystems).

**Table 1:** Primers used for PCR-amplification and sequencing in this study.

Gene	Primer		Sequence (5′→ 3′)	Product Size (bp)	Reference
16S rRNA	16F27	PCR	AGAGTTTGATCMTGGCTCAG	1400	Lane, 1991
	16R1492	PCR	TACGGYTACCTTGTTACGACTT		Lane, 1991
	16F357	Sequencing	ACTCCTACGGGAGGCAGCAG		Lane, 1991
	16R518	Sequencing	CGTATTACCGCGGCTGCTGG		Lane, 1991
*gyrB*	gyrB1F	PCR	ACAACGGCCGCGGSATTCC	1020	Tayeb *et al.*, 2008*
	UgyrBR	PCR	GCNGGRTCYTTYTCYTGRCA		Yamamoto *et al.*, 2000
	gyrBF433	Sequencing	ACAATGGCGTSAAGATCCGC		This study
	gyrBR599	Sequencing	AGCTGTCGTTCCACTGCATCG		This study
*rpoB*	rpoB-F	PCR	NGGCGAAATGGCDGARAACC	1040	Tayeb *et al.*, 2008*
	rpoB-R	PCR	NNGARTCYTCGAAGTGGTAACC		Tayeb *et al.*, 2008*
	rpoBF404	Sequencing	GTACGGCTTCCTGGAAACGC		This study
	rpoBR607	Sequencing	GCAMGGCACGGCCTGGCG		This study
*recA* #	recAF126	PCR	NCAGATYGARAAGCAGTTTYGG	770	This study
	recAF196	Sequencing	AGGTNGTNTCSACSGGNTCGC		This study
	recAR928	PCR	RCCGYYRTAGSYRTACCASGC		This study
	recAR1015	PCR	CGCGNAYNYKRTTYTCGATCTC		This study
*atpD*	atpDF30	PCR	YTTCTTGGCCTTYTCGAAGGC	900	This study
	atpDF63	Sequencing	CCGACCATGTAGAASGCCTG		This study
	atpDR1130	Sequencing	GCATCATGGACGTGCTSGG		This study
	atpDR1172	PCR	GGCRMNCCGATYTCGGTGCC		This study

*This primer has been modified from the original primer.

# For *recA* gene, the primer combination recAF126 and recAR928 was used. When the PCR failed, recAF126 and recAR1015 combination was used.

### Sequence analysis

Sequences obtained for each of the genes analyzed were assembled, manually corrected and compared to publically available sequences in Genbank, using the BLAST (Basic Local Alignment Search Tool) algorithm of the NCBI (National Center for Biotechnology Information) [36]. Alignments were performed by a hierarchical multiple alignment method implemented in the program Clustal X [37]. Sequences aligned automatically were checked manually. The evolutionary distances derived from the pair-wise differences between sequences (Jukes-Cantor correction, [38]) were calculated, using the program DNADIST, included in the phylogenetic inference package (PHYLIP 3.69) [39]. Cluster analyses and phylogenetic trees were constructed, using the Neighbor-joining distance method. Bootstrap analyses, with 1,000 repeats, were performed, using the PHYLIP program. Bootstrap values greater than 500 are indicated in the respective trees. The topologies of phylogenetic trees were visualized with the program TreeView [40]. In addition to individual cluster analyses determined for each gene, a tree derived from the concatenated sequences of the protein-coding genes was also constructed to compare the robustness of the *Achromobacter* intra-generic branching order. Similarity matrices were calculated, using the Bionumerics, version 7 software (Applied Maths NV, Sint-Martens-Latem, Belgium), and evolutionary reconstruction analyses were conducted, using the MEGA 5 program [41].

### Allele diversity and polymorphic sites

Allele diversity and polymorphic sites were calculated with the DnaSP package, version 3.51 [42].

### Nucleotide sequence accession numbers

The nucleotide sequences determined in this study have been deposited in the EMBL database under the following accession numbers: 16S rRNA gene, HG423398 to HG423445; *atpD*, HG454790 to HG454847; *gyrB*, HG454848 to HG454905; *recA*, HG454906 to HG454963 and *rpoB*, HG454964 to HG455021. GeneBank accession numbers for the sequences of the strains used in the study are available in Table S1.

### MALDI-TOF MS analyses

Matrix-Assisted Laser Desorption/Ionization-Time-Of-Flight mass spectrometry (MALDI-TOF MS) profiles for the strains studied were performed at Anagnostec, GmbH, Germany [30] and at the CCUG and the Department of Clinical Microbiology, Sahlgrenska University Hospital. Strain biomass were analyzed on a Flexi Mass stainless steel target, using a whole-cell protocol with 1 µL matrix solution of saturated α-cyano-4 hydroxy-cinnamic acid in a mixture of acetonitrile:ethanol:water (1∶1∶1) acidified with 3% (v/v) trifluoroacetic acid. For each strain, mass spectra were prepared in duplicate and analyzed on an AXIMA Confidence instrument (Shimadzu/Kratos, Manchester, UK), in the linear positive ion extraction mode. Mass spectra were accumulated from 100 profiles, each from five nitrogen laser pulse cycles, by scanning the entire sample spot. Ions were accelerated with pulsed extraction at a voltage of 20 kV. Raw mass spectra were processed automatically for baseline correction and peak recognition. Resulting mass fingerprints were exported to the SARAMIS (Spectral Archiving and Microbial Identification System, Release 3.36, Anagnostec GmbH, Germany) analysis program and compared to reference spectra. The percentage similarities of identical mass peaks were calculated and used to generate dendrograms, applying single-linkage agglomerate calculations. The spectra strains were also analyzed using the VITEK MS IVD (bioMérieux, Inc.) version 2.0.

### Phenotypic profiling

Phenotypic characterizations were performed on all strains, using customized protocols of the CCUG Typing Laboratory (http://www.ccug.se/default.cfm?navID=160) for Gram-negative, non-fermentative bacteria. The various tests included the API commercial strips for physiological profiling, API 20NE and API ZYM (bioMérieux, Inc.), and other metabolic and morphological features (Table S2).

### DNA-DNA hybridization

Total genomic DNA was isolated using the Wizard Genomic DNA Purification Kit (Promega), following the manufacturer’s instructions, and DNA-DNA hybridizations were performed, in duplicate, using a non-radioactive method described previously [43]. Reference DNA of *A. marplatensis* CCUG 56371^T^ and *A. spiritinus* CCUG 61988^T^ were double-labeled with DIG-11-dUTP and Biotin-16-dUTP, using a Nick Translation Kit (Boehringer, Mannheim, Germany). Labeled DNA was hybridized with the DNA of *A. marplatensis* CCUG 56371^T^, *A. marplatensis* CCUG 61988^T^, *A. ruhlandii* CCUG 57103^T^, *A. xylosoxidans* CCUG 56438^T^ and *A. spiritinus* strains CCUG 61969 and CCUG 61970.

## Results

### Multi-locus sequence analysis

An MLSA assessment of five selected house-keeping genes has been carried out for the *Achromobacter* species and applied to 46 strains of clinical and environmental origin. The sequences of the 16S rRNA gene, *atpD*, *gyrB*, *recA*, and *rpoB* were analyzed for the type strains of 11 validly published species of the genus. Partial sequences of the 16S rRNA genes, including nucleotide positions 28 to 500 (*Escherichia coli* 16S rRNA gene sequence numbering) were extracted from the nearly-complete sequences and the partial-sequence similarities determined and compared with those of the nearly-complete gene sequences. The 16S rRNA gene sequence similarities between the type strains of the species ranged from 99.1% to 100% for single-primer partial sequences (472 nucleotide positions) and from 99.0% to 100% for the nearly complete gene sequences (Figure S1a in File S1). The differences between the pair-wise similarities of the partial 16S rRNA gene sequences and the nearly complete gene sequences of the type strains of the *Achromobacter* species differed by an average of only 0.24%. Thus, the partial 16S rRNA gene sequences, using a single primer reaction for determining the sequences of the 5′-region of the gene, provided pair-wise similarities that are comparable with the similarities determined from nearly complete sequence comparisons. However, the problem with using 16S rRNA gene sequences is the inherently low level of resolution that exists between the sequences of the different species. Consequently, cluster analysis of the *Achromobacter* species, based upon their 16S rRNA gene sequences, exhibited little or no delineation between the species and was not further used on the MLSA study.

In the cases of protein-coding gene comparisons, the respective similarity tables were generated showing the ranges of similarities between the nucleotide sequences and also for the translated amino acid sequences of the species (Figures S2–S5 in File S1). Alignment and comparative sequence analyses of the selected house-keeping genes, *atpD*, *gyrB*, *recA,* and *rpoB*, exhibited lower levels of similarity and the respective gene trees showed greater discrimination than the 16S rRNA gene sequences between the species of *Achromobacter* (Figures S2–S5 in File S1). The sequence similarities for the house-keeping genes between the type strains of the *Achromobacter* species ranged from 94.6% to 99.3% for *atpD* (Figure S2a in File S1), 91.7% to 98.5% for *gyrB* (Figure S3a in File S1), 89.9% to 97.9% for *recA* (Figure S4a in File S1), and 92.1% to 99.8% for *rpoB* (Figure S5a in File S1). The branching orders of the sequences of the different house-keeping genes showed a high degree of consistency, *i.e*., the most closely related species were observed to be the same in all the genes studied (Figures S2b–S5b in File S1). The type strains of two pairs of species: *A. xylosoxidans* CCUG 56438^T^ and *A. ruhlandii* CCUG 57103^T^; and *A. marplatensis* CCUG 56371^T^ and *A. spiritinus* CCUG 61968^T^ exhibited the highest pair-wise similarities in all comparative house-keeping gene sequence analyses.

**Figure 1:**
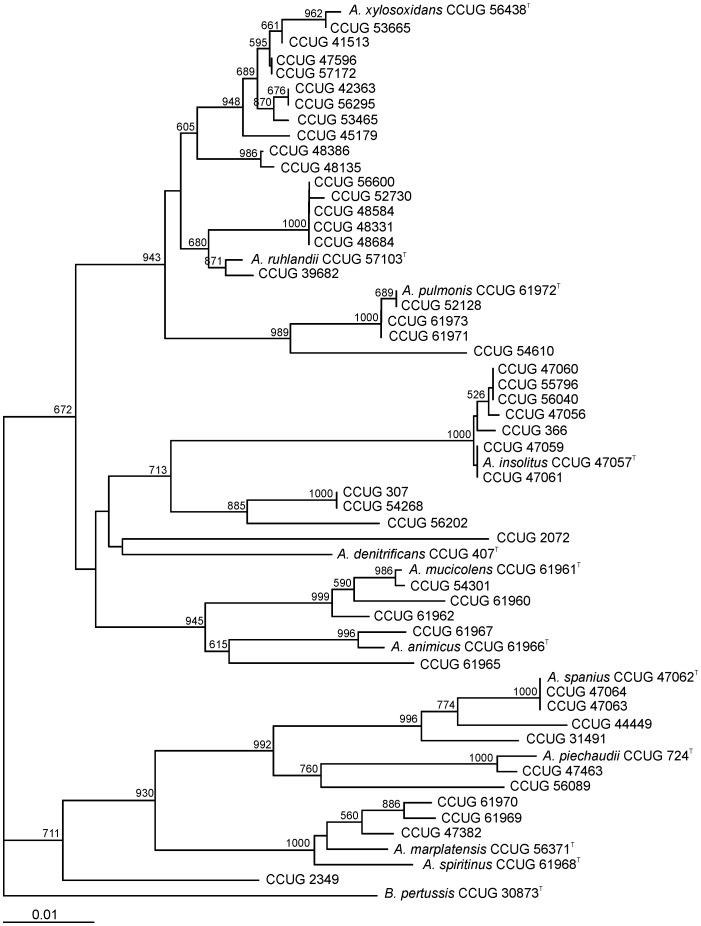
Phylogenetic tree of the strains of *Achromobacter* used in this study based on the phylogenetic analysis of *recA* gene. Distance matrix was calculated by the Jukes-Cantor method. Dendrogram was generated by neighbor-joining. *Bordetella pertussis* CCUG 30873^T^ was used as an outgroup. The bar indicates sequence divergence. Bootstrap values of more than 500 (from 1000 replicates) are indicated at the nodes.

Forty-six strains (Table S1) that had been isolated from clinical and environmental samples and identified prior to this study as *Achromobacter* species were analyzed by 16S rRNA gene and by house-keeping gene sequence comparisons. Using the sequencing primer, 16R518 (Table 1), 472 nucleotide positions of the 16S rRNA gene were used for initial estimations of taxonomic identities. For the house-keeping gene sequences, the sequencing primers (Table 1) used determined: 621 nucleotide positions for *recA*; 528 nucleotide positions for *gyrB*; 527 nucleotide positions for *rpoB*; and 513 nucleotide positions for *atpD*. The pair-wise sequence similarities determined between the clinical strains and the type strains of the *Achromobacter* species ranged from 97.6% to 100% for the 16S rRNA gene, 92.4% to 100% for *atpD*, 89.7% to 100% for *gyrB*, 87.4% to 100% for *recA*, and 91.1% to 100% for *rpoB*. Individual sequence similarities and evolutionary distances were calculated and dendrograms from the cluster analyses were generated for *recA* (Figure 1) and for each other house-keeping gene (Figures S6–S8 in File S1). The *recA*, as well as *atpD*, *gyrB*, and *rpoB* cluster analyses demonstrated greater discrimination than the 16S rRNA gene tree (Figure S9 in File S1) for the species in the *Achromobacter* genus. A concatenated analysis of *atpD*, *gyrB*, *recA* and *rpoB* genes was also performed (Figure S10 in File S1).

The individual protein-coding gene dendrograms showed high levels of congruence in the branching order of the species. Overall, the *recA* dissimilarities were greater than those observed for the other three genes assessed in this study, providing the best discrimination between species of *Achromobacter* and, in all cases, clear inter-species delineations, except for *A. marplatensis* and *A. spiritinus.* The *recA* pair-wise similarities between the type strains of the species ranged from 89.9% (between *A. piechaudii* and *A. pulmonis* and between *A. spanius* and *A. pulmonis*) to 97.6% and 97.9% (between *A. ruhlandii* and *A. xylosoxidans* and between *A. marplatensis and A. spiritinus*, respectively).

Some of the clinical and environmental strains tested were closely related to one of the species type strains (Table 2), but others (7 strains) did not cluster closely to the type strain of any described species, with similarities below the highest *recA* similarity noted for the pair-wise similarities between the type strains of the most closely related species, probably representing novel species. The same result was observed when cluster analyses derived from concatenated gene sequences were considered. High bootstrap values for the branching points confirmed the robustness of the gene sequence trees. Comparative analyses of translated nucleotide sequences to amino acid sequences for the genes studied showed that the topologies of the cluster analyses were maintained, although calculated dissimilarities were lower than those observed for nucleotide sequences.

**Table 2:** Genotypic analyses and individual *recA*- and concatenated MLSA-based identifications of clinical and environmental strains of *Achromobacter* species.

	Concatenated MLSA		*recA* analysis	
Strain	Sequence similarity# (%)	Closest species match or speciesassignation	Sequence similarity (%)	Closest species match or speciesassignation*
CCUG 307*	96.8	*A. denitrificans*	95.3	*A. denitrificans*
CCUG 366	99.4	*A. insolitus*	99.7	*A. insolitus*
CCUG 2072*	95.6	*A. denitrificans*	93.6	*A. denitrificans*
CCUG 2349*	95.6	*A. ruhlandii*	94.5	*A. ruhlandii*
CCUG 31491	98.7	*A. spanius*	97.9	*A. spanius*
CCUG 39682	99.3	*A. ruhlandii*	99.5	*A. ruhlandii*
CCUG 41513	99.5	*A. xylosoxidans*	99.4	*A. xylosoxidans*
CCUG 42363	98.8	*A. xylosoxidans*	98.7	*A. xylosoxidans*
CCUG 44449	98.7	*A. spanius*	97.9	*A. spanius*
CCUG 45179	98.9	*A. xylosoxidans*	98.3	*A. xylosoxidans*
CCUG 47056	99.6	*A. insolitus*	99.7	*A. insolitus*
CCUG 47059	99.8	*A. insolitus*	100	*A. insolitus*
CCUG 47060	99.5	*A. insolitus*	99.8	*A. insolitus*
CCUG 47061	99.8	*A. insolitus*	100	*A. insolitus*
CCUG 47063	100	*A. spanius*	100	*A. spanius*
CCUG 47064	100	*A. spanius*	100	*A. spanius*
CCUG 47382^I^	99.2/99.0	*A. spiritinus/A. marplatensis*	98.2/98.0	*A. marplatensis/A. spiritinus*
CCUG 47463	99.5	*A. piechaudii*	99.3	*A. piechaudii*
CCUG 47596	99.3	*A. xylosoxidans*	99.2	*A. xylosoxidans*
CCUG 48135	96.9	*A. ruhlandii*	98.2	*A. ruhlandii*
CCUG 48331	98.6	*A. xylosoxidans*/*A. ruhlandii*	98.5	*A. ruhlandii*
CCUG 48386	97.6	*A. ruhlandii*	98.4	*A. ruhlandii*
CCUG 48584	98.6	*A. xylosoxidans*/*A. ruhlandii*	98.5	*A. ruhlandii*
CCUG 48684	98.6	*A. xylosoxidans*/*A. ruhlandii*	98.5	*A. ruhlandii*
CCUG 52128	99.9	*A. pulmonis*	100	*A. pulmonis*
CCUG 52730	98.6	*A. ruhlandii*	98.4	*A. ruhlandii*
CCUG 53465	98.6	*A. xylosoxidans*	98.7	*A. xylosoxidans*
CCUG 53665	99.7	*A. xylosoxidans*	99.8	*A. xylosoxidans*
CCUG 54268*	96.7	*A. denitrificans*	95.3	*A. denitrificans*
CCUG 54301	97.7	*A. mucicolens*	99.8	*A. mucicolens*
CCUG 54610*	97.2	*A. ruhlandii*	96.9	*A. pulmonis*
CCUG 55796	99.7	*A. insolitus*	99.8	*A. insolitus*
CCUG 56040	99.6	*A. insolitus*	99.8	*A. insolitus*
CCUG 56089*	96.7	*A. spanius*	95.5	*A. piechaudii*
CCUG 56202*	96.4	*A. denitrificans*	95.2	*A. denitrificans*
CCUG 56295	98.7	*A. xylosoxidans*	98.7	*A. xylosoxidans*
CCUG 56600	98.6	*A. xylosoxidans*/*A. ruhlandii*	98.5	*A. ruhlandii*
CCUG 57172	99.6	*A. xylosoxidans*	99.2	*A. xylosoxidans*
CCUG 61960	98.6	*A. mucicolens*	98.5	*A. mucicolens*
CCUG 61962	99.0	*A. mucicolens*	98.5	*A. mucicolens*
CCUG 61965*	97.6	*A. animicus*	95.9	*A. animicus*
CCUG 61967	98.9	*A. animicus*	99.2	*A. animicus*
CCUG 61969^I^	99.2/99.1	*A. spiritinus/A. marplatensis*	98.7/98.2	*A. marplatensis/A. spiritinus*
CCUG 61970^I^	99.1/98.8	*A. spiritinus/A. marplatensis*	98.4/97.9	*A. marplatensis/A. spiritinus*
CCUG 61971	99.9	*A. pulmonis*	99.8	*A. pulmonis*
CCUG 61973	99.9	*A. pulmonis*	99.8	*A. pulmonis*

*indicates novel species, *i.e*., with *recA* similarities <97.6% to a recognized species.

# determined from the concatenated sequences of *atpD*, *gyrB*, *recA* and *rpoB*.

I Vandamme *et al.*, 2013a described *A. spiritinus* as a species, distinct from *A. marplatensis*.

### Determination of gene sequence polymorphic sites

The number of polymorphic sites and the allele diversity for the house-keeping genes were determined for the 57 strains included in this study (Table S3). The number of polymorphic sites in the four protein-coding loci studied ranged from 70 for *atpD* gene to 151 for *recA* gene. The *recA* gene sequence was the most discriminating of the genes analyzed, with the highest average number of nucleotide differences.

In order to compare the results of this study with those obtained in two other studies that described DNA sequence-based methods for the analyses of *Achromobacter* species [25], [29], the number of polymorphic sites for the gene sequence regions analyzed in those studies, *i.e*., *atpD*, *recA* and *rpoB*, were compared for the type strains of eleven validly published species (Table S4). For *atpD*, *recA* and *rpoB*, different regions of the gene sequences were analysed and, in all three cases, the sequence regions analysed in this study contained more polymorphic sites, providing potential for higher degrees of discrimination between species. Results were also compared with *nrdA* gene sequences (Table S4), proposed as a single locus sequencing tool for *Achromobacter* speciation (Spilker *et al.*, 2013). The short and long *nrdA* sequences exhibited 60 and 115 polymorphic sites, respectively; and 27.6 and 50.4 average number of nucleotide differences, respectively.

### MALDI-TOF MS analyses

**Figure 2:**
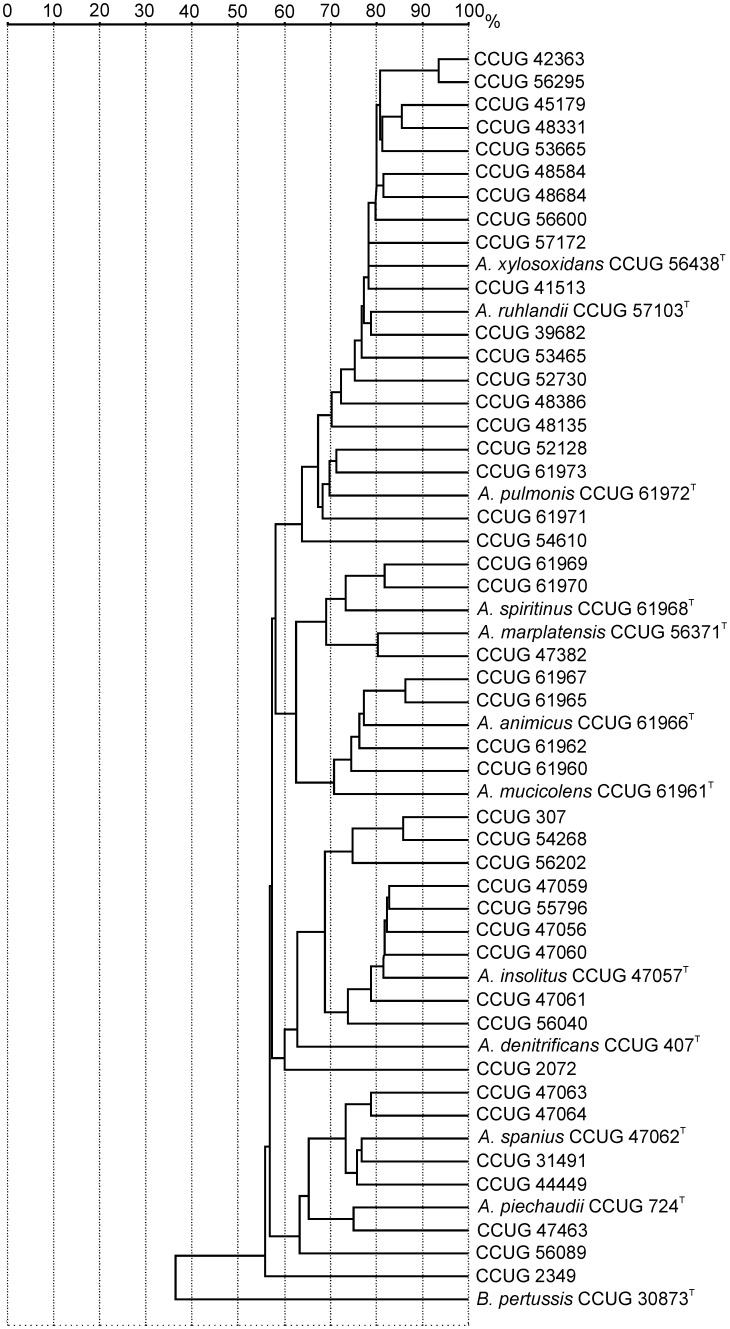
Dendogram of relatedness between the *Achromobacter* species strains analyzed based on MALDI-TOF MS analysis.

The type strains of the species of *Achromobacter* and the strains of *Achromobacter* species analyzed in this study were all identified as ‘*A. denitrificans*/*A. xylosoxidans*’, using the VITEK MS IVD Database (bioMérieux, Inc.) as reference. However, using the SARAMIS Database (bioMérieux, Inc.) [30], the MALDI-TOF MS mass signal profiles of the type strains of the eleven species were distinct (Figure 2). The MALDI-TOF MS branching order of the species in *Achromobacter* was observed to be different from that derived from the *recA* gene and the concatenated sequences of the house-keeping gene MLSA (Figure 1 and Figure S10 in File S1). However, in the cases of all *Achromobacter* species, the most closely related species determined by MALDI-TOF MS was observed to be the closest related species also by MLSA. The strains clustering with the type strains of a given species, by MALDI-TOF MS profiling, *i.e.,* at 70% similarity delineation, were observed to cluster, in most cases, with the type strain of the respective species by concatenated house-keeping gene MLSA, as well as by single-gene *recA* cluster analysis. The closely related species, *A. xylosoxidans* and *A. ruhlandii* showed a close relationship by MALDI-TOF MS analysis, with *A. ruhlandii* branching outside of the cluster of the *A. xylosoxidans* type strain and other strains clustering with *A. xylosoxidans* by MLSA, albeit with similarities lower than 70%. *A. marplatensis* and *A. spiritinus* grouped closely together by MALDI-TOF MS analysis, in two delineated branches, with a similarity at approximately 70%.

The VITEK MS IVD database (bioMérieux, Inc.) for bacterial identifications does not yet include all species of the *Achromobacter* genus; the only identifications possible for most strains of *Achromobacter* species has been an identification of ‘*A. denitrificans*/*A. xylosoxidans*’.

### Phenotypic characterization

The colonies of all strains grown on nutrient agar were whitish in color, small, *i.e*., 1 to 2 mm in diameter, mucoid, nonhemolytic, catalase positive and oxidase positive. All strains were analyzed by API ZYM and API 20NE metabolic profiling and by a series of customized tests that have been applied by the CCUG Typing Lab (www.ccug.se). The phenotypic analyses did not allow consistent, effective differentiation characterization of the strains of a given species from those of other species (Table S2).

### DNA-DNA hybridization analyses

In all cases of the genes analyzed, the type strains of *A. marplatensis* (CCUG 56371^T^) and *A. spiritinus* (CCUG 61968^T^) possessed the highest pair-wise similarities among the recognized species (98.8% concatenated sequence similarities and 97.9% in the case of *recA* gene). Such high sequence similarities for the *A. marplatensis-A. spiritinus* type strains pairs, when all other type strains of *Achromobacter*, except *A. ruhlandii* and *A. xylosoxidans*, exhibited significantly lower pair-wise sequence similarities, suggested the possibility of misclassification of *A. spiritinus*. Further analyses, including DNA-DNA hybridization (DDH), were done to confirm and clarify the taxononomic affiliation of *A. spiritinus*. The DDH similarity between *A. marplatensis* and *A. spiritinus* showed values greater than 80%, confirming that *A. spiritinus* should be considered to be the same species as *A. marplatensis*. DDH similarity values between *A. marplatensis* and the types strains used as controls, *A. ruhlandii* and *A. xylosoxidans*, were lower than 44%; when *A. spiritinus* was labelled, the values were lower than 55%. These data, were not in accordance with the recent description and valid publication of *A. spiritinus* sp. nov. [16] and led us to contact the curators of the *Achromobacter* PubMLST database, where the MLST profiles of both species were re-examined. The results of re-examination of the MLST data concluded that the original MLST data for the *A. marplatensis* type strain was incorrect, such that the MLST data of *A. spiritinus*, determined later, was not recognised to be indistinct. The MLST data for the *A. marplatensis* type strain has been corrected in the PubMLST database and these sequence data have been made available to the public (http://pubmlst.org/achromobacter/).

## Discussion

**Figure 3:**
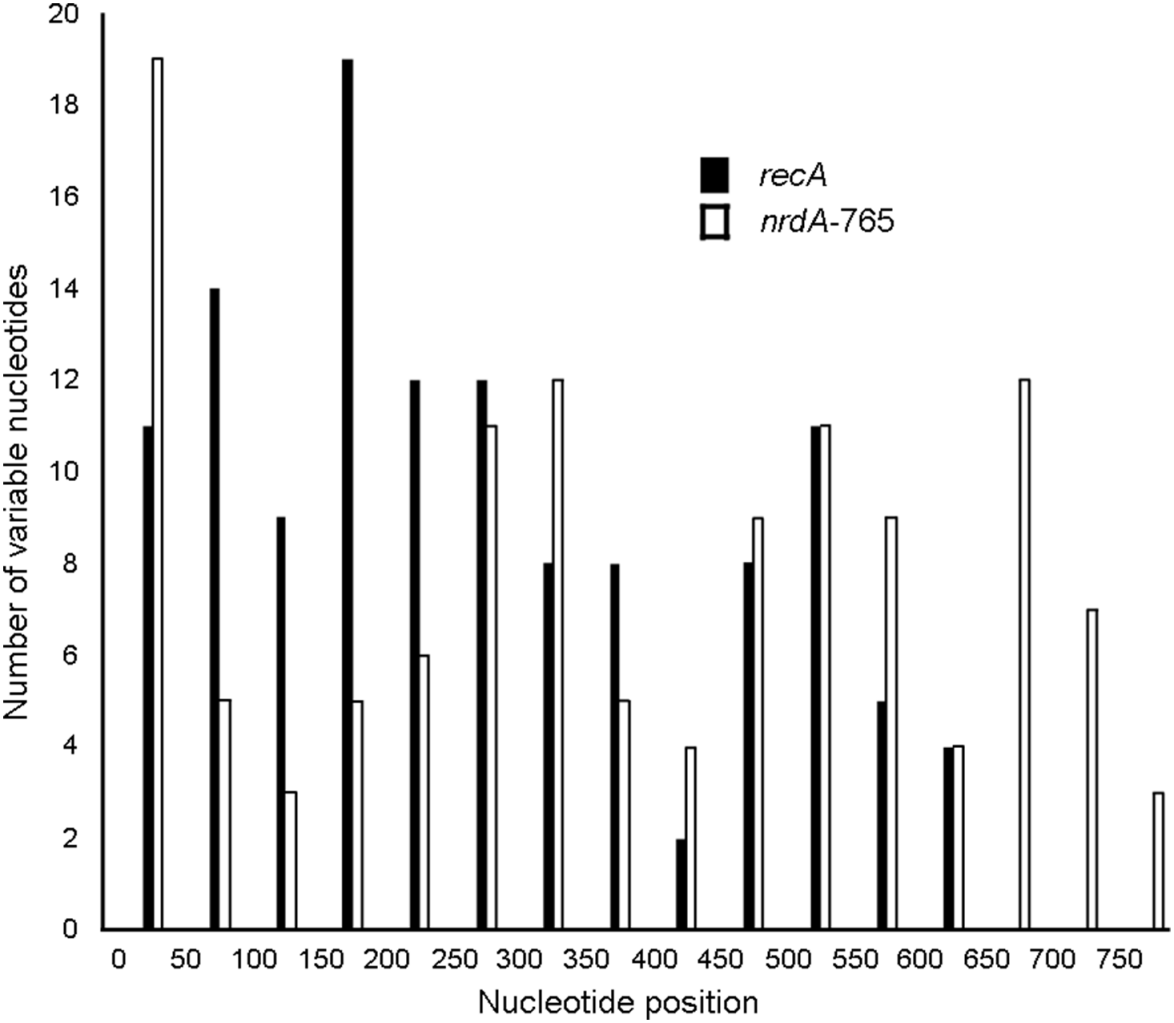
The variation in *recA* and *nrdA*-765 sequences among *Achromobacter* species. The y-axis shows the number of nucleotide positions, within 50-nucleotide position intervals (x-axis), in the respective sequences that exhibit variation between the type strains of the species of *Achromobacter*.

The MLSA in this study assessed selected house-keeping gene targets that could be applied as biomarkers for identification of the species of the genus *Achromobacter*. In the course of this study, two different research groups, in independent studies, described MLST and MLSA approaches for high-resolution discrimination of strains of individual species (*i.e*., by MLST) and the differentiation of *Achromobacter* species (*i.e*., by MLSA) [25], [29]. Spilker *et al.* (2013) assessed the ability of *nrdA* sequence analysis, one of the seven genes described in the previous MLSA scheme, to differentiate *Achromobacter* species [6], [25]. However, these studies did not focus on defining a protocol that could be applied for the reliable, rapid and cost-effective differentiation and identification of clinically-relevant strains of *Achromobacter* species for the diagnoses of infections. Our analyses have shown that *recA* sequence analyses provide a higher resolution tool for the identification of *Achromobacter* species. It is clear from studies reported in the literature and from the results of this study that traditional metabolic profile-based phenotypic testing does not allow for consistent differentiation of the species of *Achromobacter* [12], [15], [16], [25]. Furthermore, comparative sequence analysis of 16S rRNA genes, *i.e*., partial sequences or nearly complete gene sequences, possess limited value for differentiating and identifying *Achromobacter* species, due to the inherent high degrees of similarity between the sequences of the different species. Thus, additional biomarker targets of functionally-conserved, ‘house-keeping’ gene sequences, such as *atpD*, *gyrB*, *recA* and *rpoB*, offer potential, alternative species-level identification tools, since they are present in all species of the genus and they exhibit degrees of variation higher than what is observed for the 16S rRNA genes. The focus of this study was to elucidate a single house-keeping gene that would prove applicable for the delineation and identification of all species of the genus *Achromobacter* and develop a reliable, rapid and cost-effective protocol based upon comparative DNA sequence analyses. The results obtained from sequence alignments and similarity determinations demonstrated that the RecA gene has the most discriminatory sequence with the highest degree of inter-species variation. The branching order of the *Achromobacter* species, derived from cluster analyses, is maintained, for the most part, for all individual gene sequence analyses, as well as a concatenated analysis of the four protein-coding genes analyzed in this study (*i.e*., *atpD*, *gyrB*, *recA*, and *rpoB*). However, *recA* was observed to provide the greatest degree of discrimination between the most closely related species. The *recA* pair-wise similarities between the type strains of the *Achromobacter* species ranged from 89.9% to 97.6%. The species pair, *A. marplatensis* CCUG 56371^T^ compared with *A. spiritinus* CCUG 61968^T^, possessed the highest pair-wise similarities among the recognized species (97.9%), suggesting the possibility of misclassifications between them. Further DDH analysis and MLSA sequencing confirmed and clarified the taxonomic affiliation of *A. spiritinus* as a later synonym of *A. marplatensis*. From these determinations, it could be concluded that isolates and strains can be identified to the species level if they exhibit *recA* similarities of 98.0%, or greater, to a recognized species. If *recA* similarities between isolates and strains are 97.6%, or lower, to all known species, the identification will be inconclusive. The sequence data were compared also with the gene sequence data for NrdA (*nrdA*), proposed as a single locus sequencing tool for *Achromobacter* speciation by Spilker *et al.*, 2013 (Figure S11 in File S1 and Figure 3) [6]. Both genes allowed good discrimination between *Achromobacter* species with similar pair-wise similarities among them, although *recA* showed a higher number of polymorphic sites than *nrdA*.

According to the cluster analyses derived from the individual, as well as the concatenated gene sequences, the type strains of the species formed stable lineages within the genus, providing the reference points for closely related strains, while other strains were seen to diverge from the known species, representing probable new species in *Achromobacter*.

The dendrogram derived from MALDI-TOF MS mass peak profiles (Figure 2) shows species clusters similar to the ones obtained when comparing individual or concatenated house-keeping gene sequences. Considering the genotypic-based phylogeny as the basis for microbial systematics and taxonomy, the phenotypic-based MALDI-TOF MS analyses were observed to be more accurate for identifications of strains than the traditional phenotypic characterization. The species identifications, using MALDI-TOF MS and the commercially available databases, to date, have not included all of the species of the *Achromobacter* genus. The VITEK MS IVD database (bioMérieux, Inc.) contains only the species *A. xylosoxidans* and *A. denitrificans*, while the MALDI Biotyper database (Bruker Corp.) includes *A. insolitus*, *A. spanius*, *A. piechaudii* and *A. ruhlandii*, as well. In previous studies comparing the performance of MALDI-TOF MS, comparative 16S rRNA gene sequence analysis was used as the reference method, which, as previously mentioned, does not exhibit resolution adequate for definitive species-level identifications [44], [45]. The protein mass peak profile similarities between bacterial species of a genus indicates that the spectra combined with species-specific protein peaks are able to delineate most species of *Achromobacter* from each other, using MALDI-TOF MS. In the case of this study, the MALDI-TOF MS analyses of the *Achromobacter* species provided an alternative method that could be correlated with the resolution of the *Achromobacter* species by selected house-keeping gene sequence analyses.

Complete genome sequence analyses are considered to be the ultimate method for deriving the phylogenetic relationships of bacterial taxa; phylogeny is recognised to be the reference backbone of bacterial taxonomy. However, until genome sequencing is established as routine in microbiology laboratories, complementary methods are needed that provide accurate, rapid and cost-effective identifications for clinical diagnostics, biotechnology applications and environmental studies. In conclusion, selected house-keeping gene sequence analyses, *i.e*., comparative *recA* analysis is a robust method for deriving reliable identifications of clinically-relevant isolates and strains of the species in the genus *Achromobacter*. Because an exact *recA* similarity ‘cut-off’ for species identifications is not absolutely defined from sequence analyses alone, the results from alternative methods, such as MALDI-TOF MS analyses, may be correlated with the sequence data to provide insight into definitive *recA* similarity values that can be used for species-level identification.

## Supporting Information

Table S1 Strains of *Achromobacter* species and GenBank accession numbers for the sequences used in this study. Accession numbers indicated in bold are for sequences determined in this study.
(PDF)

Table S2 Biochemical characteristics of all *Achromobacter* species strains examined. +, positive; –, negative; w, weak.
(PDF)

Table S3 Genetic diversity of the selected loci among the *Achromobacter* type strains and the clinical isolates analyzed in this study.
(PDF)

Table S4 Genetic diversity values for the loci *atpD*, *recA* and *rpoB* obtained in our study compared with the results obtained for different authors, and genetic diversity for *nrdA* gene analysed for other authors. For those genetic diversity calculations only the seven type strains commons in all studies were considered.
(PDF)

File S1 Contains the following files: **Figure S1.** Gene sequence similarities and evolutionary relationships for the type strains of the *Achromobacter* species. (a) 16S rRNA gene sequence similarities for the type strains of the *Achromobacter* species. Nearly-complete 16S rRNA gene sequence similarities are in the lower diagonal; partial 16S rRNA gene sequence similarities are in the upper diagonal. (b) Evolutionary distances were computed using the Jukes-Cantor method and are in the units of the number of base substitutions per site. The analysis involved 11 nucleotide sequences. All positions containing gaps and missing data were eliminated. There were a total of 1346 positions in the final dataset. **Figure S2.** Gene sequence similarities and evolutionary relationships for the type strains of the *Achromobacter* species. (a) *atpD* gene sequence similarities for the type strains of the *Achromobacter* species gene sequence similarities are on the lower diagonal; amino acid sequence similarities are on the upper diagonal. (b) The sequence relationships were inferred, using the UPGMA method. The analysis involved 11 nucleotide sequences. All positions containing gaps and missing data were eliminated. There were a total of 727 positions in the final dataset. **Figure S3.** Gene sequence similarities and evolutionary relationships for the type strains of the *Achromobacter* species. (a) *gyrB* gene sequence similarities for the type strains of the *Achromobacter* species gene sequence similarities are on the lower diagonal; aminoacid sequence similarities are on the upper diagonal. (b) The sequence relationships were inferred, using the UPGMA method. The analysis involved 11 nucleotide sequences. All positions containing gaps and missing data were eliminated. There were a total of 593 positions in the final dataset. **Figure S4.** Gene sequence similarities and evolutionary relationships for the type strains of the *Achromobacter* species. (a) *recA* gene sequence similarities for the type strains of the *Achromobacter* species gene sequence similarities are on the lower diagonal; aminoacid sequence similarities are on the upper diagonal. (b) The sequence relationships were inferred, using the UPGMA method. The analysis involved 11 nucleotide sequences. All positions containing gaps and missing data were eliminated. There were a total of 621 positions in the final dataset. **Figure S5.** Gene sequence similarities and evolutionary relationships for the type strains of the *Achromobacter* species. (a) *rpoB* gene sequence similarities for the type strains of the *Achromobacter* species gene sequence similarities are on the lower diagonal; aminoacid sequence similarities are on the upper diagonal. (b) The sequence relationships were inferred, using the UPGMA method. The analysis involved 11 nucleotide sequences. All positions containing gaps and missing data were eliminated. There were a total of 598 positions in the final dataset. **Figure S6.** Phylogenetic tree of the 57 *Achromobacter* strains studied based on the analysis of 513 bp of the *atpD* gene. Distance matrix was calculated by the Jukes-Cantor method. Dendrogram was generated by neighbor-joining. *Bordetella pertussis* CCUG 30873^T^ was used as an outgroup. The bar indicates sequence divergence. Bootstrap values of more than 500 (from 1000 replicates) are indicated at the nodes. **Figure S7.** Phylogenetic tree of the 57 *Achromobacter* strains studied based on the analysis of 528 bp of the *gyrB* gene. Distance matrix was calculated by the Jukes-Cantor method. Dendrogram was generated by neighbor-joining. *Bordetella pertussis* CCUG 30873^T^ was used as an outgroup. The bar indicates sequence divergence. Bootstrap values of more than 500 (from 1000 replicates) are indicated at the nodes. **Figure S8.** Phylogenetic tree of the 57 *Achromobacter* strains studied based on the analysis of 527 bp of the *rpoB* gene. Distance matrix was calculated by the Jukes-Cantor method. Dendrogram was generated by neighbor-joining. *Bordetella pertussis* CCUG 30873^T^ was used as an outgroup. The bar indicates sequence divergence. Bootstrap values of more than 500 (from 1000 replicates) are indicated at the nodes. **Figure S9.** Phylogenetic tree of the 57 *Achromobacter* strains studied based on the analysis of 398 bp of the 16S rRNA gene. Distance matrix was calculated by the Jukes-Cantor method. Dendrogram was generated by neighbor-joining. *Bordetella pertussis* CCUG 30873^T^ was used as an outgroup. The bar indicates sequence divergence. Bootstrap values of more than 500 (from 1000 replicates) are indicated at the nodes. **Figure S10.** Phylogenetic tree of the strains of *Achromobacter* used in this study based on the phylogenetic analysis of four concatenated genes (*atpD*, *gyrB*, *recA* and *rpoB*). Distance matrices were calculated by the Jukes-Cantor method. Dendrograms were generated by neighbor-joining. *Bordetella pertussis* CCUG 30873^T^ was used as an outgroup. The bar indicates sequence divergence. Bootstrap values of more than 500 (from 1000 replicates) are indicated at the nodes. **Figure S11.** Gene sequence similarities and evolutionary relationships for the type strains of the *Achromobacter* species. (a) *nrdA* gene sequence similarities of a 765 pb region for the type strains of the *Achromobacter* species gene sequence similarities are on the lower diagonal; *nrdA* gene sequence similarities of the short region are on the upper diagonal.
(PDF)

## References

[1] LiPuma JJ (2010) The changing microbial epidemiology in cystic fibrosis. Clin Microbiol Rev 23:299–323. 20375354 10.1128/CMR.00068-09 PMC2863368

[2] Peleg AY, Hooper DC (2010) Hospital-acquired infections due to Gram-negative bacteria. N Engl J Med 362:1804–1813. 20463340 10.1056/NEJMra0904124 PMC3107499

[3] Amoureux L, Bador J, Fardeheb S, Mabille C, Couchot C, et al (2013) Detection of *Achromobacter xylosoxidans* in hospital, domestic, and outdoor environmental samples and comparison with human clinical isolates. Appl Environ Microbiol 79:7142–7149. 24038696 10.1128/AEM.02293-13 PMC3837737

[4] De Baets F, Schelstraete P, Van Daele S, Haerynck F, Vaneechoutte M (2007) *Achromobacter xylosoxidans* in cystic fibrosis: prevalence and clinical relevance. J Cyst Fibros 6:75–78. 16793350 10.1016/j.jcf.2006.05.011

[5] Barrado L, Brañas P, Orellana MA, Martínez MT, García G, et al (2013) Molecular characterization of *Achromobacter* isolates from cystic fibrosis and non-cystic fibrosis patients in Madrid, Spain. J Clin Microbiol 51:1927–1930. 23536401 10.1128/JCM.00494-13 PMC3716108

[6] Spilker T, Vandamme P, LiPuma JJ (2013) Identification and distribution of *Achromobacter* species in cystic fibrosis. J Cyst Fibros 12:298–301. 23141756 10.1016/j.jcf.2012.10.002

[7] De Baets F, Schelstraete P, Haerynck F, Van Bierviet S, DeBruyne R, et al (2013) *Achromobacter xylosoxidans* induced bronchiolitis obliterans in cystic fibrosis. Pediatr Pulmonol; :doi:10.1002/ppul.22864.. 10.1002/ppul.22864 24039244

[8] Hansen CR, Pressler T, Ridderberg W, Johansen HK, Skov M (2013) *Achromobacter* spcies in cystic fibrosis: cross-infection caused by indirect patient-to-patient contact. J Cyst Fibros 6:609–615. 10.1016/j.jcf.2013.05.004 23769270

[9] De Ley J, Segers P, Kersters K, Mannheim W, Lievens A (1986) Intra- and intergeneric similarities of the *Bordetella* ribosomal ribonucleic acid cistrons: proposal for a new family, *Alcaligenaceae* . Int J Syst Bacteriol 36:405–414.

[10] Yabuuchi E, Kawamura Y, Kosako Y, Ezaki T (1998) Emendation of genus *Achromobacter* and *Achromobacter xylosoxidans* (Yabuuchi and Yano) and proposal of *Achromobacter ruhlandii* (Packer and Vishniac) comb. nov., *Achromobacter piechaudii* (Kiredjian, *et al*.) comb. nov., and *Achromobacter xylosoxidans* subsp. *denitrificans* (Rüger and Tan) comb. nov. Microbiol Immunol 42:429–438. 9688077 10.1111/j.1348-0421.1998.tb02306.x

[11] Blümel S, Mark B, Busse HJ, Kämpfer P, Stolz A (2001) *Pigmentiphaga kullae* gen. nov., sp. nov., a novel member of the family *Alcaligenaceae* with the ability to decolorize azo dyes aerobically. Int J Syst Evol Microbiol 51:1867–1871. 11594620 10.1099/00207713-51-5-1867

[12] Busse HJ, Auling G (2005) Genus II. *Achromobacter* Yabuuchi and Yano 1981, 477VP emend. Yabuuchi, Kawamura, Kosako and Ezaki 1998a, 1083. In:Brenner DJ, Krieg NR, Staley JTeditors. Bergey’s Manual of Systematic Bacteriology, vol. 2: The Proteobacteria, second edition. Springer. pp. 658–662.

[13] Coenye T, Vancanneyt M, Cnockaert MC, Falsen E, Swings J, et al (2003a) *Kerstersia gyiorum* gen. nov., sp. nov., a novel *Alcaligenes faecalis*-like organism isolated from human clinical samples, and reclassification of *Alcaligenes denitrificans* Rüger and Tan 1983 as *Achromobacter denitrificans* comb. nov. Int J Syst Evol Microbiol 53:1825–1831. 14657111 10.1099/ijs.0.02609-0

[14] Coenye T, Vancanneyt M, Falsen E, Swings J, Vandamme P (2003b) *Achromobacter insolitus* sp. nov. and *Achromobacter spanius* sp. nov., from human clinical samples. Int J Syst Evol Microbiol 53:1819–1824. 14657110 10.1099/ijs.0.02698-0

[15] Gomila M, Tvrzová L, Teshim A, Sedlácek I, González-Escalona N, et al (2011) *Achromobacter marplatensis* sp. nov., isolated from a pentachlorophenol-contaminated soil. Int J Syst Evol Microbiol 61:2231–2237. 20952547 10.1099/ijs.0.025304-0

[16] Vandamme P, Moore ERB, Cnockaert M, De Brandt E, Svensson-Stadler L, et al (2013a) *Achromobacter animicus* sp. nov., *Achromobacter mucicolens* sp. nov., *Achromobacter pulmonis* sp. nov. and *Achromobacter spiritinus* sp. nov., from human clinical samples. Syst Appl Microbiol 36:1–10. 23219252 10.1016/j.syapm.2012.10.003

[17] Vandamme P, Moore ERB, Cnockaert M, Peeters C, Svensson-Stadler L, et al (2013b) Classification of *Achromobacter* genogroups 2, 5, 7 and 14 as *Achromobacter insuavis* sp. nov., *Achromobacter aegrifaciens* sp. nov., *Achromobacter anxifer* sp. nov. and *Achromobacter dolens*, sp. nov., respectively. *Syst App Microbiol* 36:474–482. 10.1016/j.syapm.2013.06.005 23891345

[18] D’Amato RF, Salemi M, Mathews A, Cleri DJ, Reddy G (1988) *Achromobacter xylosoxidans (Alcaligenes xylosoxidans* subsp. *xylosoxidans*) meningitis associated with a gunshot wound. J Clin Microbiol 26:2425–2426. 3235669 10.1128/jcm.26.11.2425-2426.1988 PMC266906

[19] Duggan JM, Goldstein SJ, Chenoweth CE, Kauffman CA, Bradley SF (1996) *Achromobacter xylosoxidans* bacteremia: report of four cases and review of the literature. Clin Infect Dis 23:569–576. 8879782 10.1093/clinids/23.3.569

[20] Liu L, Coenye T, Burns JL, Whitby PW, Stull TL, et al (2002) Ribosomal DNA-Directed PCR for identification of *Achromobacter* (*Alcaligenes*) *xylosoxidans* recovered from sputum samples from cystic fibrosis patients. J Clin Microb 40:1210–1213. 10.1128/JCM.40.4.1210-1213.2002 PMC140369 11923333

[21] Spear JB, Fuhrer J, Kirby BD (1988) *Achromobacter xylosoxidans* (*Alcaligenes xylosoxidans* subsp. *xylosoxidans*) bacteremia associated with a well-water source; case report and review of the literature. J Clin Microbiol 26:598–599. 3281982 10.1128/jcm.26.3.598-599.1988 PMC266344

[22] Vu-Thien H, Darbord JC, Moissenet D, Dulot C, Dufourcq JB, et al (1998) Investigation of an outbreak of wound infections due to *Alcaligenes xylosoxidans* transmitted by chlorhexidine in a burn unit. Eur J Clin Microbiol Infect Dis 17:724–726. 9865987 10.1007/s100960050168

[23] Kersters K, De Ley J (1984) Genus *Alcaligenes* Castellani and Chalmers 1919, 936^AL^. In:Krieg NR, Holt JGeditors. Bergey’s Manual of Systematic Bacteriology, vol. 1. Baltimore: Williams & Wilkins. pp. 361–373.

[24] Kiredjian M, Holmes B, Kersters K, Guilvout I, De Ley J (1986) *Alcaligenes piechaudii*, a new species from human clinical specimens and the environment. Int J Syst Bacteriol 36:282–287.

[25] Spilker T, Vandamme P, Lipuma JJ (2012) A multilocus sequence typing scheme implies population structure and reveals several putative novel *Achromobacter* species. J Clin Microbiol 50:3010–3015. 22785192 10.1128/JCM.00814-12 PMC3421806

[26] Wayne LG, Brenner DJ, Colwell RR, Grimont PAD, Kandler O, et al (1987) Report of the ad hoc committee on reconciliation of approaches to bacterial systematics. Int J Syst Bacteriol 37:463–464.

[27] Stackebrandt E, Frederiksen W, Garrity GM, Grimont PAD, Kämpfer P, et al (2002) Report of the *ad hoc* committee for the re-evaluation of the species definition in bacteriology. Int J Syst Evol Microbiol 52:1043–1047. 12054223 10.1099/00207713-52-3-1043

[28] Hanage WP, Fraser C, Spratt BG (2006) Sequences, sequence clusters and bacterial species. Philos Trans R Soc Lond B Biol Sci 361:1917–1927. 17062411 10.1098/rstb.2006.1917 PMC1764932

[29] Ridderberg W, Wang M, Nørskov-Lauritsen N (2012) Multilocus sequence analysis of isolates of *Achromobacter* from patients with cystic fibrosis reveals infecting species other than *Achromobacter xylosoxidans* . J Clin Microbiol 50:2688–2694. 22675125 10.1128/JCM.00728-12 PMC3421494

[30] Kallow W, Erhard M, Shah HN, Raptakis E, Welker M (2010) MALDI TOF MS for microbial identification: years of experimental development to an established protocol. In:Shah HN, Gharbia SE, Encheva Veditors. Mass Spectrometry for Microbial Proteomics, John Wiley & Sons, London. pp. 255–276.

[31] Seng P, Drancourt M, Gouriet F, La Scola B, Fournier PE, et al (2009) Ongoing revolution in bacteriology: routine identification of bacteria by matrix-assisted laser desorption ionization time-of-flight mass spectrometry. Clin Infect Dis 49:543–551. 19583519 10.1086/600885

[32] Gomila M, Ramirez A, Lalucat J (2007) Diversity of environmental *Mycobacterium* isolates from hemodialysis water as shown by a multigene sequencing approach. Appl Environ Microbiol 73:3787–3797. 17449684 10.1128/AEM.02934-06 PMC1932725

[33] Lane DJ (1991) Nucleic acid techniques in bacterial systematics. In:Stackebrand E, Goodfellow Meditors. 16S/23S rDNA sequencing. Wiley, Chichester, United Kingdom. pp. 115–175.

[34] Tayeb LA, Lefevre M, Passet V, Diancourt L, Brisse S, et al (2008) Comparative phylogenies of *Burkholderia*, *Ralstonia*, *Comamonas*, *Brevundimonas* and related organisms derived from *rpoB, gyrB* and *rrs* gene sequences. Res Microbiol 159:169–177. 18280706 10.1016/j.resmic.2007.12.005

[35] Yamamoto S, Kasai H, Arnold DL, Jackson RW, Vivian A, et al (2000) Phylogeny of the genus *Pseudomonas*: intrageneric structure reconstructed from the nucleotide sequences of *gyrB* and *rpoD* genes. Microbiology 146:2385–2394. 11021915 10.1099/00221287-146-10-2385

[36] Altschul SF, Gish W, Miller W, Myers EW, Lipman DJ (1990) Basic local alignment search tool. J Mol Biol 215:403–410. 2231712 10.1016/S0022-2836(05)80360-2

[37] Thompson JD, Gibson TJ, Plewniak F, Jeanmougin F, Higgins DG (1997) The Clustal X Windows interface: flexible strategies for multiple sequence alignment aided by quality analysis tools. Nucl Acids Res 25:4876–4882. 9396791 10.1093/nar/25.24.4876 PMC147148

[38] Jukes TH, Cantor CR (1969) Evolution of protein molecules. In:Munro HNeditors. Mammalian protein metabolism. Academic Press, Inc., New York, NY. pp. 21–132.

[39] Felsenstein J (1989) PHYLIP – phylogeny inference package (version3.2). Cladistics 5:164–166.

[40] Page RDM (1996) TREEVIEW: an application to display phylogenetic trees on personal computers. Comput Appl Biosci 12:357–358. 8902363 10.1093/bioinformatics/12.4.357

[41] Tamura K, Peterson D, Peterson N, Stecher G, Nei M, et al (2011) MEGA5: Molecular evolutionary genetics analysis using Maximum Likelihood, Evolutionary Distance, and Maximum Parsimony methods. Mol Biol Evol 28:2371–2379. 21546353 10.1093/molbev/msr121 PMC3203626

[42] Rozas J, Rozas R (1999) DnaSP version 3: an integrated program for molecular population genetics and molecular evolution analysis. Bioinformatics 15:174–175. 10089204 10.1093/bioinformatics/15.2.174

[43] Ziemke F, Hofle MG, Lalucat J, Rossello-Mora R (1998) Reclassification of *Shewanella putrefaciens* Owen’s genomic group II as *Shewanella baltica* sp. nov. Int J Syst Bacteriol 48:179–186. 9542087 10.1099/00207713-48-1-179

[44] Fernández-Olmos A, García-Castillo M, Morosini MI, Lamas A, Máiz L, et al (2012) MALDI-TOF MS improves routine identification of non-fermenting Gram negative isolates from cystic fibrosis patients. J Cyst Fibros 11:59–62. 21968086 10.1016/j.jcf.2011.09.001

[45] Jacquier H, Carbonnelle E, Corvec S, Illiaquer M, Le Monnier A, et al (2011) Revisited distribution of nonfermenting Gram-negative bacilli clinical isolates. Eur J Clin Microbiol Infect Dis 30:1579–1586. 21509476 10.1007/s10096-011-1263-5

