# Supplementary material for: Genotypic and Phenotypic Applications for the Differentiation and Species-Level Identification of Achromobacter for Clinical Diagnoses
Source: PLoS One. 2014 Dec 4;9(12):e114356. doi: 10.1371/journal.pone.0114356 (PMC4256396; doi:10.1371/journal.pone.0114356)
Supplement: Table S1 — Strains of Achromobacter species and GenBank accession numbers for the sequences used in this study. Accession numbers indicated in bold are for sequences determined in this study. (PDF) [file pone.0114356.s001.pdf]

**Supplemental Table S1.** Strains of *Achromobacter* species and GenBank accession numbers for the sequences used in this study. Accession numbers indicated in bold are for sequences determined in this study.

| Strain | *recA* identification | Origin | 16SrRNA | *atpD* | *gyrB* | *recA* | *rpoB* |
|---|---|---|---|---|---|---|---|
| CCUG 407$^{T}$ | *A. denitrificans* | Soil | FM999734 | **HG454790** | **HG454848** | **HG454906** | **HG454964** |
| CCUG 724$^{T}$ | *A. piechaudii* | Pharynx | FM999731 | **HG454791** | **HG454849** | **HG454907** | **HG454965** |
| CCUG 47057$^{T}$ | *A. insolitus* | Leg wound | FM999733 | **HG454792** | **HG454850** | **HG454908** | **HG454966** |
| CCUG 47062$^{T}$ | *A. spanius* | Blood | FM999732 | **HG454793** | **HG454851** | **HG454909** | **HG454967** |
| CCUG 56371$^{T}$ | *A. marplatensis* | Soil | EU150134 | **HG454794** | **HG454852** | **HG454910** | **HG454968** |
| CCUG 56438$^{T}$ | *A. xylosoxidans* | Ear discharge | FM999735 | **HG454795** | **HG454853** | **HG454911** | **HG454969** |
| CCUG 57103$^{T}$ | *A. ruhlandii* | Soil | **HG423398** | **HG454796** | **HG454854** | **HG454912** | **HG454970** |
| CCUG 61961$^{T}$ | *A. mucicolens* | Human sputum, non-CF patient | HE613446 | **HG454797** | **HG454855** | **HG454913** | **HG454971** |
| CCUG 61966$^{T}$ | *A. animicus* | Human sputum, non-CF patient | HE613448 | **HG454798** | **HG454856** | **HG454914** | **HG454972** |
| CCUG 61968$^{T}$ | *A. spiritinus* | Human sputum, non-CF patient | HE613447 | **HG454799** | **HG454857** | **HG454915** | **HG454973** |
| CCUG 61972$^{T}$ | *A. pulmonis* | Human sputum, CF patient | HE798552 | **HG454800** | **HG454858** | **HG454916** | **HG454974** |
| CCUG 307* | *A. denitrificans* | Human wound, 18-yr-old man | **HG423399** | **HG454801** | **HG454859** | **HG454917** | **HG454975** |
| CCUG 366 | *A. insolitus* | Human pleural fluid | **HG423400** | **HG454802** | **HG454860** | **HG454918** | **HG454976** |
| CCUG 2072* | *A. denitrificans* | Not known | **HG423401** | **HG454803** | **HG454861** | **HG454919** | **HG454977** |
| CCUG 2349* | *A. ruhlandii* | Human blood | **HG423402** | **HG454804** | **HG454862** | **HG454920** | **HG454978** |
| CCUG 31491 | *A. spanius* | Not known | **HG423403** | **HG454805** | **HG454863** | **HG454921** | **HG454979** |
| CCUG 39682 | *A. ruhlandii* | Human, CF patient | **HG423404** | **HG454806** | **HG454864** | **HG454922** | **HG454980** |
| CCUG 41513 | *A. xylosoxidans* | Human sputum,26-yr-old patient, CF | **HG423405** | **HG454807** | **HG454865** | **HG454923** | **HG454981** |
| CCUG 42363 | *A. xylosoxidans* | Human sputum,18-yr-old male, CF | **HG423406** | **HG454808** | **HG454866** | **HG454924** | **HG454982** |
| CCUG 44449 | *A. spanius* | Human choledochus secretion, 61-yr-old woman | **HG423407** | **HG454809** | **HG454867** | **HG454925** | **HG454983** |
| CCUG 45179 | *A. xylosoxidans* | Human sputum, CF | **HG423408** | **HG454810** | **HG454868** | **HG454926** | **HG454984** |
| CCUG 47056 | *A. insolitus* | Laboratory sink | **HG423409** | **HG454811** | **HG454869** | **HG454927** | **HG454985** |

| | | | | | | | |
|---|---|---|---|---|---|---|---|
| CCUG 47059 | *A. insolitus* | Human wound | **HG423410** | **HG454812** | **HG454870** | **HG454928** | **HG454986** |
| CCUG 47060 | *A. insolitus* | Not known | **HG423411** | **HG454813** | **HG454871** | **HG454929** | **HG454987** |
| CCUG 47061 | *A. insolitus* | Not known | **HG423412** | **HG454814** | **HG454872** | **HG454930** | **HG454988** |
| CCUG 47063 | *A. spanius* | Not known | **HG423413** | **HG454815** | **HG454873** | **HG454931** | **HG454989** |
| CCUG 47064 | *A. spanius* | Not known | **HG423414** | **HG454816** | **HG454874** | **HG454932** | **HG454990** |
| CCUG 47382 | *A. marplatensis/A. spiritinus* | Not known | **HG423415** | **HG454817** | **HG454875** | **HG454933** | **HG454991** |
| CCUG 47463 | *A. piechaudii* | Human bronchial alveolar lavage, 58-yr-old woman | **HG423416** | **HG454818** | **HG454876** | **HG454934** | **HG454992** |
| CCUG 47596 | *A. xylosoxidans* | Human CF, 21-yr-old female | **HG423417** | **HG454819** | **HG454877** | **HG454935** | **HG454993** |
| CCUG 48135 | *A. ruhlandii* | Human sputum, CF patient | **HG423418** | **HG454820** | **HG454878** | **HG454936** | **HG454994** |
| CCUG 48331 | *A. ruhlandii* | Human sputum, 29-year old woman, CF | **HG423419** | **HG454821** | **HG454879** | **HG454937** | **HG454995** |
| CCUG 48386 | *A. ruhlandii* | Human sputum, 30-yr-old woman, CF | **HG423420** | **HG454822** | **HG454880** | **HG454938** | **HG454996** |
| CCUG 48584 | *A. ruhlandii* | Human sputum, 29-yr-old woman, CF | **HG423421** | **HG454823** | **HG454881** | **HG454939** | **HG454997** |
| CCUG 48684 | *A. ruhlandii* | Human sputum,30-yr-old woman, CF | **HG423422** | **HG454824** | **HG454882** | **HG454940** | **HG454998** |
| CCUG 52128 | *A. pulmonis* | Environmental control | **HG423423** | **HG454825** | **HG454883** | **HG454941** | **HG454999** |
| CCUG 52730 | *A. ruhlandii* | Human sputum,50-yr-old woman | **HG423424** | **HG454826** | **HG454884** | **HG454942** | **HG455000** |
| CCUG 53465 | *A. xylosoxidans* | Human synovial fluid,81-yr-old man, prosthesis | **HG423425** | **HG454827** | **HG454885** | **HG454943** | **HG455001** |
| CCUG 53665 | *A. xylosoxidans* | Human sputum,89-yr-old woman | **HG423426** | **HG454828** | **HG454886** | **HG454944** | **HG455002** |
| CCUG 54268* | *A. denitrificans* | Human trachea,39-yr-old man | **HG423427** | **HG454829** | **HG454887** | **HG454945** | **HG455003** |
| CCUG 54301 | *A. mucicolens* | Human sputum,13-yr-old boy | **HG423428** | **HG454830** | **HG454888** | **HG454946** | **HG455004** |
| CCUG 54610* | *A. pulmonis* | Human eye lens,16-yr-old boy | **HG423429** | **HG454831** | **HG454889** | **HG454947** | **HG455005** |
| CCUG 55796 | *A. insolitus* | Human sputum,16-yr-old male, CF | **HG423430** | **HG454832** | **HG454890** | **HG454948** | **HG455006** |
| CCUG 56040 | *A. insolitus* | Human sputum,16-yr-old male, CF | **HG423431** | **HG454833** | **HG454891** | **HG454949** | **HG455007** |
| CCUG 56089* | *A. piechaudii* | Human mucosa,75-yr-old man, cheek | **HG423432** | **HG454834** | **HG454892** | **HG454950** | **HG455008** |
| CCUG 56202* | *A. denitrificans* | Human sputum,65-yr-old woman, CF | **HG423433** | **HG454835** | **HG454893** | **HG454951** | **HG455009** |
| CCUG 56295 | *A. xylosoxidans* | Human sputum,27-yr-old man | **HG423434** | **HG454836** | **HG454894** | **HG454952** | **HG455010** |
| CCUG 56600 | *A. ruhlandii* | Human sputum,34-yr-old woman, CF | **HG423435** | **HG454837** | **HG454895** | **HG454953** | **HG455011** |
| CCUG 57172 | *A. xylosoxidans* | Human bronchioalveolar lavage,50-yr-old man | **HG423436** | **HG454838** | **HG454896** | **HG454954** | **HG455012** |
| CCUG 61960 | *A. mucicolens* | Human epiglottis swab, non-CF patient | **HG423437** | **HG454839** | **HG454897** | **HG454955** | **HG455013** |

| | | | | | | | |
|---|---|---|---|---|---|---|---|
| CCUG 61962 | *A. mucicolens* | Human sputum, CF patient | **HG423438** | **HG454840** | **HG454898** | **HG454956** | **HG455014** |
| CCUG 61965* | *A. animicus* | Human sputum, CF patient | **HG423439** | **HG454841** | **HG454899** | **HG454957** | **HG455015** |
| CCUG 61967 | *A. animicus* | Human sputum, CF patient | **HG423440** | **HG454842** | **HG454900** | **HG454958** | **HG455016** |
| CCUG 61969 | *A. marplatensis/A. spiritinus* | Human sputum, non-CF patient | **HG423441** | **HG454843** | **HG454901** | **HG454959** | **HG455017** |
| CCUG 61970 | *A. marplatensis/A. spiritinus* | Human sputum, non-CF patient | **HG423442** | **HG454844** | **HG454902** | **HG454960** | **HG455018** |
| CCUG 61971 | *A. pulmonis* | Human sputum, CF patient | **HG423443** | **HG454845** | **HG454903** | **HG454961** | **HG455019** |
| CCUG 61973 | *A. pulmonis* | Human sputum, CF patient | **HG423444** | **HG454846** | **HG454904** | **HG454962** | **HG455020** |
| CCUG 30873$^T$ | *Bordetella pertussis* | Unknown | **HG423445** | **HG454847** | **HG454905** | **HG454963** | **HG455021** |

*, indicated novel species, i.e., with *recA* gene similarities <97.6% to a recognized species.
