# Supplementary material for: Genotypic and Phenotypic Applications for the Differentiation and Species-Level Identification of Achromobacter for Clinical Diagnoses
Source: PLoS One. 2014 Dec 4;9(12):e114356. doi: 10.1371/journal.pone.0114356 (PMC4256396; doi:10.1371/journal.pone.0114356)
Supplement: Table S2 — Biochemical characteristics of all Achromobacter species strains examined. +, positive; –, negative; w, weak. (PDF) [file pone.0114356.s002.pdf]

Supplemental Table S2. Biochemical characteristics of all *Achromobacter* species strains examined. +, positive; -, negative; w, weak.

		**Tests OX**									**Tests DEC**																					
**STRAIN**	***recA* IDENTIFICATION**	**Oxidase, TMPD**	**Catalase**	**Hemolysis, horse blood**	**Penicillin, mm**	**Blood 30°C - growth**	**Blood 37°C - growth**	**Drigalski - growth**	**NA 42°C - growth**	**Motility HD/30°C or OF**	**OF-D-Glucose**	**OF-Maltose**	**OF-Adonitol**	**OF-D-Fructose**	**OF-D-Xylose**	**Fluorescin**	**Centrimide**	**10% Lactose**	**Tween 80**	**Amylase, MH**	**6% NaCl, growth**	**Lysine, LD**	**Ornithine, OD**	**Arginine, ADH**	**NO$_3^-$-reduction**	**NO$_2^-$-reduction**	**N_2/N_2O-denitrification**	**Urease, Christ.**	**Gelatine Kohn**	**Esculine hydrolysis**	**ONPG**
CCUG 56438^{T}	*A. xylosoxidans*	+	+	-	-	+	+	w	+	+	-	-	-	-	-	-	+	-	-	-	-	-	-	-	+	+	+	-	-	-	-
CCUG 41513	*A. xylosoxidans*	w	+	-	-	w	+	w	w	+	+	-	-	-	w	-	-	-	-	-	-	-	-	-	+	w	+	-	-	-	-
CCUG 42363	*A. xylosoxidans*	+	+	-	-	+	+	+	+	+	-	-	-	-	w	-	+	-	-	-	-	-	-	-	-	+	-	-	-	-	-
CCUG 45179	*A. xylosoxidans*	w	+	-	-	+	+	w	+	+	-	-	-	-	+	-	+	-	-	-	-	-	-	-	-	w	-	-	-	-	-
CCUG 47596	*A. xylosoxidans*	+	+	-	-	w	+	w	+	-	-	-	-	-	-	-	+	-	-	-	-	-	-	-	+	+	+	-	-	-	-
CCUG 53465	*A. xylosoxidans*	+	+	-	-	+	+	+	+	+	-	-	-	-	-	-	+	-	-	-	-	-	-	-	+	+	-	-	-	-	-
CCUG 53665	*A. xylosoxidans*	+	+	-	-	+	+	+	+	+	-	-	-	-	w	-	+	-	-	-	-	-	-	-	+	+	+	-	-	-	-
CCUG 56295	*A. xylosoxidans*	+	w	-	-	+	+	+	+	+	-	-	-	-	-	-	+	-	-	-	-	-	-	-	+	+	+	-	-	-	-
CCUG 57172	*A. xylosoxidans*	+	+	-	-	+	+	+	+	+	-	-	-	-	-	-	+	-	-	-	w	-	-	-	+	+	+	-	-	-	-
CCUG 57103^{T}	*A. ruhlandii*	+	+	-	-	+	+	+	-	+	-	-	-	-	w	-	w	-	-	-	-	-	-	-	+	-	-	-	-	-	-
CCUG 2349*	*A. ruhlandii*	-	+	-	-	+	+	+	w	+	-	-	-	-	-	-	+	-	-	-	-	-	-	-	+	-	-	-	-	-	-
CCUG 39682	*A. ruhlandii*	+	+	-	-	+	+	w	+	w	-	-	-	-	-	w	w	-	-	-	-	-	-	-	+	-	-	-	-	-	-
CCUG 48135	*A. ruhlandii*	w	+	-	-	+	+	+	w	+	-	-	-	-	+	-	+	-	-	-	-	-	-	-	+	+	+	-	-	-	-
CCUG 48331	*A. ruhlandii*	+	+	-	-	+	+	+	+	+	-	-	-	-	+	-	+	-	-	-	-	-	-	+	+	+	+	-	-	-	-
CCUG 48386	*A. ruhlandii*	+	+	-	-	+	+	+	-	+	-	-	-	-	+	-	+	-	-	-	-	-	-	-	+	+	+	-	-	-	-
CCUG 48584	*A. ruhlandii*	+	+	-	-	+	+	+	+	+	-	-	-	-	-	-	+	-	-	-	-	-	-	-	+	+	+	-	-	-	-
CCUG 48684	*A. ruhlandii*	+	+	-	-	+	+	+	+	+	-	-	-	-	-	-	+	-	-	-	-	-	-	-	+	+	+	-	-	-	-
CCUG 52730	*A. ruhlandii*	+	+	-	-	+	+	-	+	-	-	-	-	-	w	-	+	-	-	-	-	-	-	-	+	-	-	-	-	-	-
CCUG 56600	*A. ruhlandii*	+	+	-	-	w	+	w	+	+	-	-	-	-	-	-	+	-	-	-	-	-	-	-	+	w	+	-	-	-	-
CCUG 724^{T}	*A. piechaudii*	+	+	-	-	+	+	+	-	+	-	-	-	-	-	-	+	-	-	-	-	-	-	-	+	-	-	-	-	-	-
CCUG 47463	*A. piechaudii*	+	w	-	-	+	+	+	w	+	-	-	-	-	-	-	w	-	-	-	-	-	-	-	+	-	-	-	-	-	-
CCUG 56089*	*A. piechaudii*	w	+	-	-	+	+	+	-	+	-	-	-	-	-	-	w	-	-	-	-	-	-	-	+	-	-	-	-	-	-
CCUG 407^{T}	*A. denitrificans*	+	+	-	-	+	+	+	w	+	-	-	-	-	-	-	+	-	-	-	-	-	-	-	+	+	+	-	-	-	-
CCUG 307*	*A. denitrificans*	nd	nd	w	-	+	+	+	-	+	-	-	-	-	-	-	+	-	-	-	-	-	-	-	+	+	+	-	-	-	-
CCUG 2072*	*A. denitrificans*	+	+	-	-	+	+	+	+	w	-	-	-	-	-	-	+	-	-	-	-	-	-	-	+	+	+	-	-	-	-
CCUG 56202*	*A. denitrificans*	+	+	-	-	+	+	+	-	+	-	-	-	-	-	-	+	-	-	-	-	-	-	-	+	w	-	-	-	-	-
CCUG 54268*	*A. denitrificans*	+	+	-	-	+	+	w	-	+	-	-	-	-	-	-	+	-	-	-	-	-	-	w	+	+	+	-	-	-	-
CCUG 47057^{T}	*A. insolitus*	+	+	-	-	+	+	+	+	w	-	-	-	-	-	-	-	-	-	-	-	-	-	-	+	-	-	-	-	-	-
CCUG 366	*A. insolitus*	+	+	-	-	+	+	+	+	+	-	-	-	-	-	-	+	-	-	-	-	-	-	-	+	-	-	-	-	-	-
CCUG 47056	*A. insolitus*	+	+	-	-	+	+	+	+	+	-	-	-	-	-	-	+	-	-	-	-	-	-	-	+	-	-	-	-	-	-
CCUG 47059	*A. insolitus*	+	+	-	-	+	+	+	+	+	-	-	-	-	-	-	w	-	-	-	-	-	-	-	+	-	-	-	-	-	-
CCUG 47060	*A. insolitus*	+	+	-	-	+	+	+	+	+	-	-	-	-	-	-	w	-	-	-	-	-	-	-	+	-	-	-	-	-	-
CCUG 47061	*A. insolitus*	+	+	-	-	+	+	+	+	+	-	-	-	-	-	-	w	-	-	-	-	-	-	-	+	-	-	-	-	-	-
CCUG 55796	*A. insolitus*	+	+	-	-	+	+	+	w	+	-	-	-	-	-	-	+	-	-	-	-	-	-	-	+	-	-	-	-	-	-
CCUG 56040	*A. insolitus*	+	+	-	-	+	+	w	-	+	-	-	-	-	-	-	+	-	-	-	-	-	-	-	+	-	-	-	-	-	-
CCUG 47062^{T}	*A. spanius*	w	+	-	-	+	+	+	+	+	-	-	-	-	-	-	-	-	-	-	-	-	-	-	+	-	-	-	-	-	-
CCUG 31491	*A. spanius*	+	+	-	-	w	+	w	-	-	w	-	-	-	-	-	+	-	-	-	-	-	-	-	+	-	-	w	-	-	+
CCUG 44449	*A. spanius*	w	w	-	-	w	+	w	-	+	-	-	-	-	-	-	w	-	-	-	-	w	-	-	-	-	-	-	-	-	-
CCUG 47063	*A. spanius*	+	+	-	-	+	+	+	-	+	-	-	-	-	-	-	-	-	-	-	-	-	-	-	+	-	-	w	-	-	-
CCUG 47064	*A. spanius*	+	+	-	-	+	+	w	-	+	-	-	-	-	-	-	w	-	-	-	w	-	-	-	+	-	-	-	-	-	-
CCUG 56371^{T}	*A. marplatensis*	+	+	-	-	+	+	+	-	w	-	-	-	-	-	-	w	-	-	-	-	-	-	-	+	w	-	-	-	-	-
CCUG 47382	*A. spiritinus/A. marplatensis*	w	+	-	-	+	+	+	w	+	-	-	-	-	-	-	w	-	-	-	-	-	-	-	+	w	-	-	-	-	-
CCUG 61966^{T}	*A. animicus*	+	+	-	-	+	+	+	+	+	-	-	-	-	-	-	-	-	-	-	-	-	-	-	+	+	+	-	-	-	-
CCUG 61965*	*A. animicus*	+	+	-	-	+	+	+	-	+	-	-	-	-	-	-	-	-	-	-	-	-	-	-	+	+	+	-	-	-	-
CCUG 61967	*A. animicus*	+	+	-	-	+	+	+	-	+	-	-	-	-	-	-	+	-	-	-	-	-	-	-	+	+	+	-	-	-	-
CCUG 61961^{T}	*A. mucicolens*	+	+	-	-	+	+	+	+	+	-	-	-	-	-	-	w	-	-	-	-	-	-	-	+	+	+	-	-	-	-
CCUG 54301	*A. mucicolens*	+	+	-	-	+	+	w	+	+	-	-	-	-	-	-	-	-	-	-	-	-	-	w	+	+	+	-	-	-	-
CCUG 61960	*A. mucicolens*	w	+	-	-	+	+	+	+	+	-	-	-	-	-	-	-	-	-	-	-	-	-	-	+	+	w	-	-	-	-
CCUG 61962	*A. mucicolens*	+	+	-	-	+	+	-	-	+	-	-	-	-	-	-	-	-	-	-	-	-	-	-	+	+	+	-	-	-	-
CCUG 61972^{T}	*A. pulmonis*	w	+	-	-	+	+	+	+	+	-	-	-	-	-	-	w	-	-	-	-	-	-	-	+	-	-	-	-	-	-
CCUG 52128	*A. pulmonis*	w	+	-	-	+	+	+	+	+	-	-	-	-	-	-	+	-	-	-	+	-	-	-	+	-	-	-	-	-	-
CCUG 54610*	*A. pulmonis*	+	+	-	-	+	+	+	+	-	-	-	-	-	-	-	-	w	-	-	-	-	-	w	+	-	-	-	-	-	-
CCUG 61971	*A. pulmonis*	+	+	-	-	+	+	w	+	-	-	-	-	-	-	-	+	-	-	-	-	-	-	-	+	-	-	-	-	-	-
CCUG 61973	*A. pulmonis*	+	+	-	-	+	+	+	+	+	-	-	-	-	w	-	w	-	-	-	+	-	-	-	+	-	-	-	-	-	-
CCUG 61968^{T}	*A. spiritinus*	w	+	-	-	+	+	+	+	+	-	-	-	-	-	-	-	-	-	-	-	-	-	-	+	-	-	-	-	-	-
CCUG 61969	*A. marplatensis/A. spiritinus*	+	+	-	-	+	+	+	-	+	-	-	-	-	-	-	w	-	-	-	-	-	-	-	+	-	-	-	-	-	-
CCUG 61970	*A. marplatensis/A. spiritinus*	+	+	-	-	+	+	+	-	+	-	-	-	-	-	-	w	-	-	-	-	-	-	-	+	-	-	-	-	-	-
**STRAIN**	**MLSA IDENTIFICATION**	**Oxidase, TMPD**	**Catalase**	**Hemolysis, horse blood**	**Penicillin, mm**	**Blood 30°C - growth**	**Blood 37°C - growth**	**Drigalski - growth**	**NA 42°C - growth**	**Motility HD/30°C or OF**	**OF-D-Glucose**	**OF-Maltose**	**OF-Adonitol**	**OF-D-Fructose**	**OF-D-Xylose**	**Fluorescin**	**Centrimide**	**10% Lactose**	**Tween 80**	**Amylase, MH**	**6% NaCl, growth**	**Lysine, LD**	**Ornithine, OD**	**Arginine, ADH**	**NO$_3^-$-reduction**	**NO$_2^-$-reduction**	**N_2/N_2O-denitrification**	**Urease, Christ.**	**Gelatine Kohn**	**Esculine hydrolysis**	**ONPG**
		**Tests OX**									**Tests DEC**																					

*, indicated novel species, i.e., with *recA* gene similarities <97.9% to a recognized species

Tests ESC									Tests ASSIM																		Tests API20NE														
Indole	DNAse	Acetamide	Acid in TSI	H_2S in TSI	NaCl 0.5% - growth	NaCl 1.5% - growth	NaCl 3.0% - growth	NaCl 4.5% - growth	Glucose	Trehalose	Arginine	Norleucin	Arabinose	Mannose	Mannitol	N-Ac-Glucosamine	Maltose	Gluconate	Caprate	Adipate	Malate	Citrate	Phenylacetate	Sucrose	Lactate	Lactate + Methionine	Nitrate reduction	Tryptophanase	Glucose fermentation	Arg. dihydrol	Urease	Esculin	Gelatinase	PNPG β-gal	D-Glucose	L-Arabinose	D-Mannose	D-Mannitol	N-Ac-glucosamine	Maltose	D-Gluconate
-	-	+	-	-	+	+	+	w	+	-	-	-	-	-	-	-	-	+	-	-	+	+	+	-	+	+	+	-	-	-	-	-	-	-	+	-	-	-	-	-	+
-	-	w	-	-	+	+	w	-	+	-	-	-	-	-	-	-	-	+	-	w	+	+	+	-	-	w	+	-	-	-	-	-	-	-	+	-	-	-	-	-	w
-	-	w	-	-	+	w	-	-	+	-	-	-	-	-	-	-	-	+	w	+	+	+	+	-	w	w	-	-	-	-	-	-	-	-	+	-	-	-	-	-	+
-	-	-	-	-	+	+	-	-	+	-	-	-	-	-	-	-	-	+	+	+	+	+	+	-	-	+	-	-	-	-	-	-	-	-	+	-	-	-	-	-	+
-	-	+	-	-	+	+	+	+	-	-	-	-	-	-	-	-	-	+	w	+	+	+	+	-	+	+	+	-	-	-	-	-	-	-	w	-	-	-	-	-	+
-	-	-	-	-	+	+	+	w	-	-	-	-	-	-	-	-	-	-	-	-	-	-	-	-	-	-	+	-	-	-	-	-	-	-	w	-	-	-	-	-	+
-	-	+	-	-	+	+	-	-	+	-	-	-	-	-	-	-	-	+	w	+	+	+	+	-	+	+	+	-	-	-	-	-	-	-	+	-	-	-	-	-	+
-	-	-	-	-	+	+	+	-	+	-	-	-	-	-	-	-	-	+	-	+	+	+	+	-	+	+	+	-	-	-	-	-	-	-	+	-	+	-	-	-	+
-	-	+	-	-	+	+	+	+	+	-	-	-	-	-	-	-	-	+	-	+	+	+	+	-	+	+	+	-	-	-	-	-	-	-	+	-	+	-	-	-	+
-	-	-	-	-	+	+	w	-	w	-	-	-	-	-	-	-	-	w	w	w	+	+	w	-	w	w	+	-	-	-	-	-	-	-	+	-	-	-	-	-	+
-	-	+	-	-	+	+	+	+	-	-	-	-	-	-	-	-	-	+	+	+	+	+	+	-	+	+	+	-	-	-	-	-	-	-	-	-	-	-	-	-	+
-	-	-	-	-	+	w	w	-	w	-	-	-	-	-	-	-	w	-	-	w	+	+	+	-	w	w	+	-	-	-	-	-	-	-	w	-	-	-	-	-	+
-	-	+	-	-	+	+	+	w	+	-	-	-	-	-	-	-	-	+	-	+	+	+	+	-	+	+	+	-	-	-	-	-	-	-	+	-	-	-	-	-	+
-	-	+	-	-	+	+	-	-	+	-	-	-	-	-	-	-	-	+	+	+	+	+	+	-	+	+	+	-	-	-	-	-	-	-	+	-	w	-	-	-	+
-	-	-	-	-	+	+	w	w	+	-	-	-	-	-	-	-	-	+	+	+	+	+	+	-	+	+	+	-	-	-	-	-	-	-	+	-	-	-	-	-	+
-	-	+	-	-	+	+	-	-	+	-	-	-	-	-	-	-	-	+	+	+	+	+	+	-	+	+	+	-	-	-	-	-	-	-	+	-	-	-	-	-	+
-	-	+	-	-	+	+	-	-	+	-	-	-	-	-	-	-	-	+	+	+	+	+	+	-	+	+	+	-	-	-	-	-	-	-	+	-	-	-	-	-	+
-	-	-	-	-	+	+	w	-	+	+	+	+	+	+	+	+	+	+	+	+	+	+	+	w	+	+	+	-	-	-	-	-	-	-	+	-	-	-	-	-	+
-	-	+	-	-	+	+	-	-	+	-	-	-	-	-	-	-	-	+	w	+	+	+	+	-	+	+	+	-	-	-	-	-	-	-	+	-	w	-	-	-	+
-	-	-	-	-	+	+	+	+	-	-	-	-	-	-	-	-	-	+	w	+	+	+	+	-	-	-	+	-	-	-	-	-	-	-	-	-	-	-	-	-	+
-	-	-	-	-	+	+	+	+	-	-	-	-	-	-	-	-	-	+	-	+	+	+	+	-	+	+	+	-	-	-	-	-	-	-	w	-	-	-	-	-	+
-	-	-	-	-	+	+	+	w	-	-	-	-	-	-	-	-	w	+	w	+	+	+	+	-	w	+	+	-	-	-	-	-	-	-	w	-	-	-	-	-	+
-	-	+	-	-	+	+	+	w	-	-	-	-	-	-	-	-	-	-	+	+	+	+	+	-	+	+	+	-	-	-	-	-	-	-	-	-	-	-	-	-	-
-	nd	nd	-	-	nd	+	+	-	w	-	-	-	-	-	-	-	-	+	-	+	+	+	w	-	+	+	+	-	-	-	-	-	-	-	-	-	-	-	-	-	+
-	-	+	-	-	+	+	+	+	-	-	-	-	-	-	-	-	-	-	w	w	w	+	w	-	w	+	+	-	-	-	-	-	-	-	-	-	-	-	-	-	-
-	-	+	-	-	+	+	+	w	-	-	-	-	-	-	-	-	-	+	w	+	+	+	+	-	+	+	+	-	-	-	-	-	-	-	-	-	-	-	-	-	+
-	-	+	-	-	+	+	+	+	-	-	-	-	-	-	-	-	-	+	+	+	+	+	+	-	+	+	+	-	-	-	-	-	-	-	-	-	-	-	-	-	+
-	-	w	-	-	+	+	+	w	-	-	-	-	-	-	-	-	-	+	w	+	+	+	+	-	+	+	+	-	-	-	-	-	-	-	-	-	-	-	-	-	+
-	-	+	-	-	+	+	+	w	+	-	-	-	-	-	-	-	-	+	-	+	+	+	+	-	+	+	+	-	-	-	-	-	-	-	-	-	-	-	-	-	+
-	-	+	-	-	+	+	w	w	-	-	-	w	-	-	-	-	-	+	w	+	+	+	+	-	+	+	+	-	-	-	-	-	-	-	w	-	-	-	-	-	+
-	-	+	-	-	+	+	w	w	-	-	-	-	-	-	-	-	-	w	w	w	w	+	w	-	w	+	+	-	-	-	-	-	-	-	w	-	-	-	-	-	+
-	-	w	-	-	+	+	w	w	-	-	-	-	-	-	-	-	-	+	w	+	+	+	+	-	w	w	+	-	-	-	-	-	-	-	w	-	-	-	-	-	+
-	-	w	-	-	+	+	w	w	-	-	-	-	-	-	-	-	-	w	-	w	w	w	w	-	-	w	+	-	-	-	-	-	-	-	w	-	-	-	-	-	+
-	-	+	-	-	+	+	-	-	-	-	-	-	-	-	-	-	-	+	+	+	+	+	+	-	+	+	+	-	-	-	-	-	-	-	-	-	-	-	-	-	+
-	-	+	-	-	+	+	-	-	-	-	-	w	-	w	-	w	-	+	+	+	+	+	+	-	+	+	+	-	-	-	-	-	-	-	-	-	-	-	-	-	+
-	-	-	-	-	+	+	w	w	-	-	-	-	-	-	-	-	-	w	-	-	+	+	w	-	-	w	+	-	-	-	-	-	-	-	w	w	-	-	-	-	+
-	-	-	-	-	+	+	w	-	w	w	-	-	w	w	-	w	-	w	-	w	+	+	w	-	-	w	+	-	-	-	-	-	-	-	-	-	-	-	-	-	+
-	-	-	-	-	+	+	w	-	-	-	-	-	-	-	-	-	-	+	w	+	+	+	+	-	+	+	w	-	-	-	-	-	-	-	-	-	-	-	-	-	+
-	-	-	-	-	+	+	w	w	-	-	-	-	-	-	-	-	-	w	-	-	+	+	w	-	-	w	+	-	-	-	-	-	-	-	-	-	-	-	-	-	+
-	-	-	-	-	+	+	w	w	-	-	-	-	-	-	-	-	-	w	-	-	+	+	w	-	w	w	+	-	-	-	-	-	-	-	w	-	-	-	-	-	+
-	-	-	-	-	+	+	+	-	-	-	-	w	-	-	-	-	-	+	w	+	+	+	+	-	+	+	+	-	-	-	-	-	-	-	-	-	-	-	-	-	-
-	-	-	-	-	+	+	w	w	-	-	-	-	-	-	-	-	-	w	w	w	+	+	w	-	w	w	+	-	-	-	-	-	-	-	-	-	-	-	-	-	+
-	-	-	-	-	+	+	+	w	-	-	-	-	-	-	-	-	-	+	w	+	+	+	+	-	-	+	+	-	-	-	-	-	-	-	-	-	-	-	-	-	+
-	-	w	-	-	+	+	+	-	-	-	-	-	-	-	-	-	-	+	w	+	+	+	+	-	-	+	+	-	-	-	-	-	-	-	-	-	-	-	-	-	+
-	-	-	-	-	+	+	+	-	-	-	-	-	-	-	-	-	-	+	w	+	+	+	+	-	w	+	+	-	-	-	-	-	-	-	-	-	-	-	-	+	+
-	-	-	-	-	+	+	+	w	-	-	-	w	-	-	-	-	-	+	w	+	+	+	+	-	+	+	+	-	-	-	-	-	-	-	-	-	-	-	-	-	+
-	-	-	-	-	+	+	w	-	-	-	-	-	-	-	-	-	-	w	-	+	+	+	+	-	w	+	+	-	-	-	-	-	-	-	-	-	-	-	-	-	+
-	-	-	-	-	+	+	+	-	-	-	-	-	-	-	-	-	-	+	+	+	+	+	+	-	-	+	+	-	-	-	-	-	-	-	-	-	-	-	-	-	+
-	-	-	-	-	+	+	+	w	-	-	-	w	-	-	-	-	-	+	+	+	+	+	+	-	+	+	+	-	-	-	-	-	-	-	-	-	-	-	-	-	+
-	-	-	-	-	+	+	w	-	-	-	-	-	-	-	-	-	-	+	+	+	+	+	+	-	-	w	+	-	-	-	-	-	-	-	-	-	-	-	-	-	+
-	-	w	-	-	+	+	+	+	-	-	-	-	-	-	-	-	-	+	+	+	+	+	+	-	+	+	-	-	-	-	-	-	-	-	-	-	-	-	-	-	+
-	-	+	-	-	+	+	+	w	-	-	-	-	-	-	-	-	-	+	-	+	+	+	+	-	+	+	+	-	-	-	-	-	-	-	-	-	-	-	-	-	+
-	-	-	-	-	+	+	+	w	-	-	-	-	-	-	-	-	-	-	-	-	-	-	-	-	-	-	+	-	-	-	-	-	-	-	-	-	-	-	-	-	w
-	-	+	-	-	+	+	+	+	+	-	-	-	-	-	-	-	-	+	+	+	+	+	+	-	+	+	+	-	-	-	-	-	-	-	+	-	-	-	-	-	+
-	-	-	-	-	+	+	w	-	-	-	-	w	-	-	-	-	-	+	w	+	+	+	+	-	+	+	+	-	-	-	-	-	-	-	-	-	-	-	-	-	w
-	-	-	-	-	+	+	+	w	-	-	-	-	-	-	-	-	-	+	+	+	+	+	+	-	+	+	+	-	-	-	-	-	-	-	-	-	-	-	-	-	+
-	-	-	-	-	+	+	+	-	-	-	-	-	-	-	-	-	-	+	+	+	+	+	+	-	+	+	+	-	-	-	-	-	-	-	-	-	-	-	-	-	w

Indole DNAse Acetamide Acid in TSI H_2S in TSI NaCl 0.5% - growth NaCl 1.5% - growth NaCl 3.0% - growth NaCl 4.5% - growth Glucose Trehalose Arginine Norleucin Arabinose Mannose Mannitol N-Ac-Glucosamine Maltose Gluconate Caprate Adipate Malate Citrate Phenylacetate Sucrose Lactate Lactate + Methionine Nitrate reduction Tryptophanase Glucose fermentation Arg. dihydrol Urease Esculin Gelatinase PNPG β-gal D-Glucose L-Arabinose D-Mannose D-Mannitol N-Ac-glucosamine Maltose D-Gluconate

Tests ESC Tests ASSIM Tests API20NE

						Tests API ZYM																			
**Caprate**	**Adipate**	**L-Malate**	**Citrate**	**Phenylacetate**	**Oxidase**	**Alkaline Phosphatase**	**Esterase (C-4)**	**Ester Lipase (C-8)**	**Lipase (C-14)**	**Leucine arylamidase**	**Valine arylamidase**	**Cysteine arylamidase**	**Trypsin**	**Chymotrypsin**	**Acid Phosphatase**	**Phosphoamidase**	**α-galactosidase**	**β-galactosidase**	**β-glucuronidase**	**α-glucosidase**	**β-glucosidase**	**N-Acetyl-β-gluc**	**α-mannosidase**	**α-fucosidase**	**STRAIN**
+	+	+	+	+	+	w	w	-	-	+	-	-	-	w	+	-	-	-	-	-	-	-	-	-	CCUG 56438T
-	w	+	w	w		w	w	w	-	+	-	-	-	+	+	-	-	-	-	-	-	-	-	-	CCUG 41513
w	w	+	+	+	+	-	-	-	-	+	-	-	-	-	+	-	-	-	-	-	-	-	-	-	CCUG 42363
+	w	+	+	w		-	+	-	-	+	-	-	-	-	-	-	-	-	-	-	-	-	-	-	CCUG 45179
+	+	+	+	+	+	+	+	-	-	+	-	-	-	-	+	-	-	-	-	-	+	-	-	-	CCUG 47596
w	w	+	+	+	+	w	+	-	-	+	-	-	-	w	w	-	-	-	-	-	-	-	-	-	CCUG 53465
+	+	+	+	+	+	+	+	-	-	+	-	-	-	-	+	-	-	-	-	-	-	-	-	-	CCUG 53665
+	+	+	+	+	+	w	w	-	-	+	-	-	-	-	+	-	-	-	-	-	-	-	-	-	CCUG 56295
+	+	+	+	+	+	w	w	-	-	+	-	-	-	-	+	-	-	-	-	-	-	-	-	-	CCUG 57172
w	w	+	+	+	+	-	-	-	-	+	-	-	-	-	w	-	-	-	-	-	-	-	-	-	CCUG 57103T
+	+	+	+	+	-	+	-	-	-	+	-	-	-	w	+	-	-	-	-	-	-	-	-	-	CCUG 2349
w	w	+	+	+	+	w	w	w	-	+	-	-	-	-	+	-	-	-	-	-	-	-	-	-	CCUG 39682
-	-	+	+	+	w	+	w	-	-	+	-	-	-	-	+	-	-	-	-	-	-	-	-	-	CCUG 48135
+	+	+	+	+	+	w	+	-	-	+	-	-	-	-	+	-	-	-	-	-	-	-	-	-	CCUG 48331
+	+	+	+	+	+	+	w	-	-	+	-	-	-	-	+	-	-	-	-	-	-	-	-	-	CCUG 48386
+	+	+	+	+	+	w	+	-	-	+	-	-	-	-	+	-	-	-	-	-	-	-	-	-	CCUG 48584
+	+	+	+	+	+	w	+	-	-	+	-	-	-	-	+	-	-	-	-	-	-	-	-	-	CCUG 48684
w	-	+	+	+	+	w	w	-	-	+	-	-	-	-	+	-	-	-	-	-	-	-	-	-	CCUG 52730
+	+	+	+	+	+	-	w	-	-	+	-	-	-	-	+	-	-	-	-	-	-	-	-	-	CCUG 56600
-	+	+	+	+	+	-	+	-	-	+	-	-	-	-	-	-	-	-	-	-	-	-	-	-	CCUG 724T
-	+	+	+	+	+	w	w	-	-	+	-	-	-	-	+	-	-	-	-	-	-	-	-	-	CCUG 47463
w	+	+	+	+	w	-	-	-	-	+	-	-	-	-	+	-	-	-	w	-	-	-	-	-	CCUG 56089
+	+	+	+	+	+	-	+	-	-	+	-	-	-	-	-	-	-	-	-	-	-	-	-	-	CCUG 407T
+	+	+	+	+	+	+	w	-	-	+	-	-	-	-	+	-	-	-	-	-	-	-	-	-	CCUG 307
w	+	+	+	+	+	-	w	-	-	+	-	-	-	-	-	-	-	-	-	-	-	-	-	-	CCUG 2072
+	+	+	+	+	+	-	-	-	-	+	-	-	-	-	-	-	-	-	-	-	-	-	-	-	CCUG 56202
-	-	+	+	+	+	+	+	-	-	+	-	-	-	-	+	-	-	-	-	-	-	-	-	-	CCUG 54268
w	w	+	+	+	+	w	w	-	-	+	-	-	-	-	+	-	-	-	-	-	-	-	-	-	CCUG 47057T
+	+	+	+	+	+	-	w	w	-	+	-	-	-	-	+	-	-	-	-	-	-	-	-	-	CCUG 366
w	w	+	+	+	+	w	+	-	-	+	-	-	-	-	+	-	-	-	-	-	-	-	-	-	CCUG 47056
+	+	+	+	+	+	w	+	-	-	+	-	-	-	-	+	-	-	-	-	-	-	-	-	-	CCUG 47059
-	+	+	+	+	+	-	w	-	-	+	-	-	-	-	-	-	-	-	-	-	-	-	-	-	CCUG 47060
-	+	+	+	+	+	-	w	-	-	+	-	-	-	-	+	-	-	-	-	-	-	-	-	-	CCUG 47061
+	+	+	+	+	+	-	-	-	-	+	-	-	-	-	+	-	-	-	-	-	-	-	-	-	CCUG 55796
+	+	+	+	+	+	w	w	-	-	+	-	-	-	-	+	-	-	-	-	-	-	-	-	-	CCUG 56040
-	-	+	+	+	w	-	+	-	-	+	-	-	-	-	w	-	-	-	-	-	-	-	-	-	CCUG 47062T
-	+	+	+	w	+	w	w	-	-	+	-	-	-	-	+	-	-	-	-	-	-	-	-	-	CCUG 31491
-	+	+	+	+	w	w	w	-	-	+	-	-	-	-	+	-	-	-	-	-	-	-	-	-	CCUG 44449
-	-	+	+	+	+	-	w	-	-	+	-	-	-	-	+	-	-	-	-	-	-	-	-	-	CCUG 47063
-	-	+	+	+	+	-	w	-	-	+	-	-	-	-	+	-	-	-	-	-	-	-	-	-	CCUG 47064
-	-	w	w	w	+	+	w	-	-	+	-	-	-	-	+	w	-	-	-	-	-	-	-	-	CCUG 56371T
w	w	+	+	+	w	w	-	-	-	+	-	-	-	-	+	-	-	-	-	-	-	-	-	-	CCUG 47382
-	+	+	+	+	+	-	w	-	-	+	-	-	-	-	-	-	-	-	-	-	-	-	-	-	CCUG 61966T
-	+	+	+	+	+	-	+	-	-	+	-	-	-	-	w	w	-	-	-	-	-	-	-	-	CCUG 61965
w	+	+	+	+	+	w	w	-	-	+	-	-	-	-	+	w	-	-	-	-	-	-	-	-	CCUG 61967
w	+	+	+	+	+	-	w	-	-	+	-	-	-	-	-	w	-	-	-	-	-	-	-	-	CCUG 61961T
w	+	+	+	+	+	-	-	-	-	w	-	-	-	-	-	-	-	-	-	-	-	-	-	-	CCUG 54301
-	+	+	+	+	w	-	+	-	-	+	w	-	-	-	-	w	-	-	-	-	-	-	-	-	CCUG 61960
w	+	+	+	+	+	-	w	-	-	+	-	-	-	-	-	-	-	-	-	-	-	-	-	-	CCUG 61962
-	+	+	+	+	w	-	-	-	-	+	-	-	-	-	-	-	-	-	-	-	-	-	-	-	CCUG 61972T
+	+	+	+	+	w	-	+	-	-	+	-	-	-	-	w	w	-	-	-	-	-	-	-	-	CCUG 52128
+	+	+	+	+	+	-	-	-	-	+	-	-	-	-	-	-	-	-	-	-	-	-	-	-	CCUG 54610
-	-	-	w	-	-	-	w	-	-	w	-	-	-	-	-	-	-	-	-	-	-	-	-	-	CCUG 61971
+	+	+	+	+	+	-	w	-	-	+	-	-	-	-	-	-	-	-	-	-	-	-	-	-	CCUG 61973
+	+	+	+	+	w	+	w	-	-	+	-	-	-	-	+	w	-	-	-	-	-	-	-	-	CCUG 61968T
w	+	+	+	+	+	w	w	-	-	+	-	-	-	-	+	-	-	-	-	-	-	-	-	-	CCUG 61969
-	w	+	+	+	+	-	w	-	-	+	-	-	-	-	+	-	-	-	-	-	-	-	-	-	CCUG 61970

Caprate Adipate L-Malate Citrate Phenylacetate Oxidase Alkaline Phosphatase Esterase (C-4) Ester Lipase (C-8) Lipase (C-14) Leucine arylamidase Valine arylamidase Cysteine arylamidase Trypsin Chymotrypsin Acid Phosphatase Phosphoamidase α-galactosidase β-galactosidase β-glucuronidase α-glucosidase β-glucosidase N-Acetyl-β-gluc α-mannosidase α-fucosidase STRAIN

Tests API ZYM
