# Supplementary material for: Genotypic and Phenotypic Applications for the Differentiation and Species-Level Identification of Achromobacter for Clinical Diagnoses
Source: PLoS One. 2014 Dec 4;9(12):e114356. doi: 10.1371/journal.pone.0114356 (PMC4256396; doi:10.1371/journal.pone.0114356)
Supplement: Table S3 — Genetic diversity of the selected loci among the Achromobacter type strains and the clinical isolates analyzed in this study. (PDF) [file pone.0114356.s003.pdf]

Supplemental Table S3. Genetic diversity of the selected loci among the *Achromobacter* type strains and the clinical isolates analyzed in this study.

No. of strains	Locus	Fragment length (bp)	No. of alleles	No. of polymorphic sites
**Type strains**				
11	16S rRNA	1354	10	13
11	*atpD*	727	11	68
11	*gyrB*	593	11	100
11	*recA*	621	11	128
11	*rpoB*	598	11	79
**Clinical isolates**				
57	16S rRNA	344	4	7
57	*atpD*	513	34	70
57	*gyrB*	528	45	124
57	*recA*	621	43	151
57	*rpoB*	527	43	89
