# Supplementary material for: Genotypic and Phenotypic Applications for the Differentiation and Species-Level Identification of Achromobacter for Clinical Diagnoses
Source: PLoS One. 2014 Dec 4;9(12):e114356. doi: 10.1371/journal.pone.0114356 (PMC4256396; doi:10.1371/journal.pone.0114356)
Supplement: Table S4 — Genetic diversity values for the loci atpD, recA and rpoB obtained in our study compared with the results obtained for different authors, and genetic diversity for nrdA gene analysed for other authors. For those genetic diversity calculations only the seven type strains commons in all studies were considered. (PDF) [file pone.0114356.s004.pdf]

Supplemental Table S4. Genetic diversity values for the loci *atpD*, *recA* and *rpoB* obtained in our study compared with the results obtained for different authors, and genetic diversity for *nrdA* gene analyzed for other authors. For those genetic diversity calculations only the seven type strains commons in all studies were considered.

	Locus	Fragment length (bp)	No. of alleles	No. of polymorphic sites	Average number of nucleotide differences
This study	*atpD*	775	7	62	26.57143
Riddeberg *et al.*		399	7	50	21.90476
This study	*recA*	695	7	124	52.09524
Riddeberg *et al.*		459	7	92	40.09524
This study	*rpoB*	964	7	104	44.42857
Riddeberg *et al.*		513	7	66	27.33333
Spilker *et al.*		413	7	30	11.42857
Spilker *et al.*	*nrdA*	449	7	60	27.61905
Spilker *et al.*	*nrdA*	765	7	115	50.38095
