# Supplementary material for: Genotypic and Phenotypic Applications for the Differentiation and Species-Level Identification of Achromobacter for Clinical Diagnoses
Source: PLoS One. 2014 Dec 4;9(12):e114356. doi: 10.1371/journal.pone.0114356 (PMC4256396; doi:10.1371/journal.pone.0114356)
Supplement: File S1 — Contains the following files: Figure S1. Gene sequence similarities and evolutionary relationships for the type strains of the Achromobacter species. (a) 16S rRNA gene sequence similarities for the type strains of the Achromobacter species. Nearly-complete 16S rRNA gene sequence similarities are in the lower diagonal; partial 16S rRNA gene sequence similarities are in the upper diagonal. (b) Evolutionary distances were computed using the Jukes-Cantor method and are in the units of the number of base substitutions per site. The analysis involved 11 nucleotide sequences. All positions containing gaps and missing data were eliminated. There were a total of 1346 positions in the final dataset. Figure S2. Gene sequence similarities and evolutionary relationships for the type strains of the Achromobacter species. (a) atpD gene sequence similarities for the type strains of the Achromobacter species gene sequence similarities are on the lower diagonal; amino acid sequence similarities are on the upper diagonal. (b) The sequence relationships were inferred, using the UPGMA method. The analysis involved 11 nucleotide sequences. All positions containing gaps and missing data were eliminated. There were a total of 727 positions in the final dataset. Figure S3. Gene sequence similarities and evolutionary relationships for the type strains of the Achromobacter species. (a) gyrB gene sequence similarities for the type strains of the Achromobacter species gene sequence similarities are on the lower diagonal; aminoacid sequence similarities are on the upper diagonal. (b) The sequence relationships were inferred, using the UPGMA method. The analysis involved 11 nucleotide sequences. All positions containing gaps and missing data were eliminated. There were a total of 593 positions in the final dataset. Figure S4. Gene sequence similarities and evolutionary relationships for the type strains of the Achromobacter species. (a) recA gene sequence similarities for the type strains of the [file pone.0114356.s005.pdf]

Supplemental Figure S1. Gene sequence similarities and evolutionary relationships for the type strains of the *Achromobacter* species. (a) 16S rRNA gene sequence similarities for the type strains of the *Achromobacter* species. Nearly-complete 16S rRNA gene sequence similarities are in the lower diagonal; partial 16S rRNA gene sequence similarities are in the upper diagonal. (b) Evolutionary distances were computed using the Jukes-Cantor method and are in the units of the number of base substitutions per site. The analysis involved 11 nucleotide sequences. All positions containing gaps and missing data were eliminated. There were a total of 1346 positions in the final dataset.

(a)

	1	2	3	4	5	6	7	8	9	10	11
1. *A. xylosoxidans*	100.0	100.0	99.2	100.0	99.8	99.4	99.4	99.1	99.4	100.0	99.2
2. *A. ruhlandii*	99.8	100.0	99.2	100.0	99.8	99.4	99.4	99.1	99.4	100.0	99.2
3. *A. piechaudii*	99.2	99.3	100.0	99.2	99.0	99.8	99.8	99.5	99.8	99.2	99.8
4. *A. denitrificans*	99.5	99.6	99.0	100.0	99.8	99.4	99.4	99.1	99.4	100.0	99.2
5. *A. insolitus*	99.5	99.6	99.3	99.5	100.0	99.2	99.2	99.2	99.2	99.8	99.0
6. *A. spanius*	99.3	99.4	99.9	99.1	99.4	100.0	100.0	99.7	100.0	99.4	99.8
7. *A. marplatensis*	99.3	99.5	99.7	99.2	99.3	99.8	100.0	99.7	100.0	99.4	99.8
8. *A. animicus*	99.5	99.6	99.3	99.3	99.4	99.4	99.4	100.0	99.7	99.1	99.5
9. *A. mucicolens*	99.4	99.4	99.4	99.2	99.3	99.5	99.4	99.6	100.0	99.4	99.8
10. *A. pulmonis*	99.6	99.6	99.0	99.4	99.3	99.0	99.1	99.4	99.2	100.0	99.2
11. *A. spiritinus*	99.1	99.3	99.5	99.2	99.2	99.6	99.8	99.2	99.2	98.9	100.0

The type strains for each species: 1. CCUG 56438^{T}; 2. CCUG 57103^{T}; 3. CCUG 724^{T}; 4. CCUG 407^{T}; 5. CCUG 47057^{T}; 6. CCUG 47062^{T}; 7. CCUG 56371^{T}; 8. CCUG 61966^{T}; 9. CCUG 61961^{T}; 10. CCUG 61972^{T}; 11. CCUG 61968^{T}.

(b)

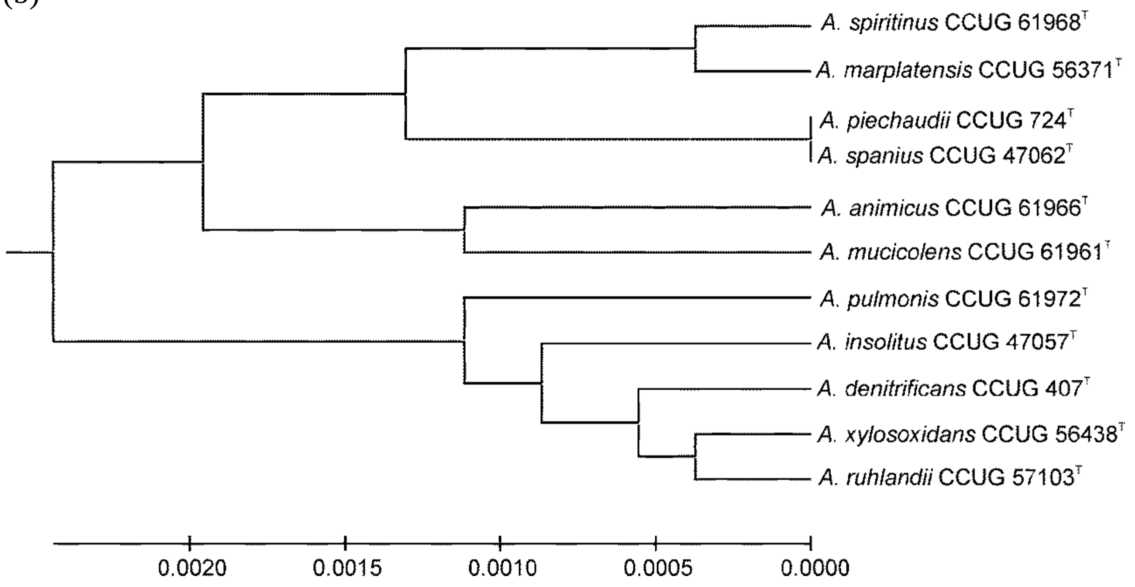

Supplemental Figure S2. Gene sequence similarities and evolutionary relationships for the type strains of the *Achromobacter* species. (a) *atpD* gene sequence similarities for the type strains of the *Achromobacter* species gene sequence similarities are on the lower diagonal; amino acid sequence similarities are on the upper diagonal. (b) The sequence relationships were inferred, using the UPGMA method. The analysis involved 11 nucleotide sequences. All positions containing gaps and missing data were eliminated. There were a total of 727 positions in the final dataset.

(a)

	1	2	3	4	5	6	7	8	9	10	11
1. *A. xylosoxidans*	100.0	100.0	99.7	100.0	100.0	99.7	99.7	99.7	100.0	100.0	99.5
2. *A. ruhlandii*	99.3	100.0	99.7	100.0	100.0	99.7	99.7	99.7	100.0	100.0	99.5
3. *A. piechaudii*	94.5	94.9	100.0	99.7	99.7	100.0	100.0	99.5	99.7	99.7	99.8
4. *A. denitrificans*	96.8	96.7	96.3	100.0	100.0	99.7	99.7	99.7	100.0	100.0	99.5
5. *A. insolitus*	97.0	97.4	96.7	97.9	100.0	99.7	99.7	99.7	100.0	100.0	99.5
6. *A. spanius*	95.2	95.6	98.1	96.0	96.2	100.0	100.0	99.5	99.7	99.7	99.8
7. *A. marplatensis*	94.8	95.2	98.1	95.9	95.9	97.4	100.0	99.5	99.7	99.7	99.8
8. *A. animicus*	95.5	96.2	96.8	95.9	96.6	96.6	96.4	100.0	99.7	99.7	99.2
9. *A. mucicolens*	96.2	96.8	97.0	96.6	97.0	96.7	96.8	97.9	100.0	100.0	99.5
10. *A. pulmonis*	98.6	98.8	94.9	96.7	97.1	95.3	95.1	95.2	96.6	100.0	99.5
11. *A. spiritinus*	94.6	95.1	97.7	95.7	95.7	97.5	99.0	96.0	96.4	94.9	100.0

The type strains for each species: 1. CCUG 56438^T; 2. CCUG 57103^T; 3. CCUG 724^T; 4. CCUG 407^T; 5. CCUG 47057^T; 6. CCUG 47062^T; 7. CCUG 56371^T; 8. CCUG 61966^T; 9. CCUG 61961^T; 10. CCUG 61972^T; 11. CCUG 61968^T.

(b)

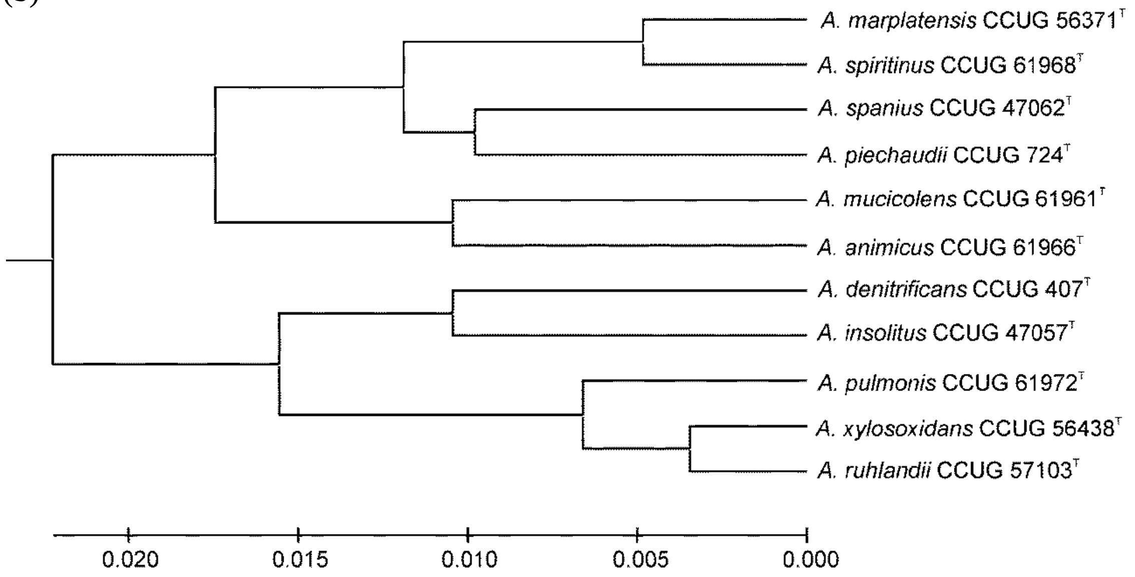

Supplemental Figure S3. Gene sequence similarities and evolutionary relationships for the type strains of the *Achromobacter* species. (a) *gyrB* gene sequence similarities for the type strains of the *Achromobacter* species gene sequence similarities are on the lower diagonal; aminoacid sequence similarities are on the upper diagonal. (b) The sequence relationships were inferred, using the UPGMA method. The analysis involved 11 nucleotide sequences. All positions containing gaps and missing data were eliminated. There were a total of 593 positions in the final dataset.

(a)

	1	2	3	4	5	6	7	8	9	10	11
1. *A. xylosoxidans*	100.0	99.7	97.5	99.0	99.4	97.2	97.1	98.7	98.7	99.0	97.5
2. *A. ruhlandii*	98.5	100.0	97.5	99.4	99.7	97.2	97.1	99.0	98.7	99.3	97.5
3. *A. piechaudii*	93.8	93.4	100.0	97.5	97.1	99.7	98.4	98.5	98.8	98.1	98.7
4. *A. denitrificans*	95.6	95.8	92.6	100.0	99.7	97.2	96.8	99.0	98.7	99.3	97.2
5. *A. insolitus*	95.1	95.3	92.6	95.8	100.0	96.9	97.1	98.7	98.4	99.0	97.5
6. *A. spanius*	93.1	92.8	96.1	92.4	91.7	100.0	98.1	98.2	98.5	97.9	98.4
7. *A. marplatensis*	94.1	93.4	94.6	93.8	93.3	94.3	100.0	97.5	97.8	97.2	99.7
8. *A. animicus*	94.3	94.3	95.0	94.1	93.6	94.6	94.8	100.0	99.7	99.7	97.8
9. *A. mucicolens*	93.8	93.9	94.1	93.9	92.9	93.3	94.1	97.1	100.0	99.4	98.2
10. *A. pulmonis*	94.4	95.3	92.8	92.9	92.6	91.9	91.7	93.3	93.8	100.0	97.5
11. *A. spiritinus*	94.3	93.6	94.3	94.1	93.6	93.9	99.0	94.8	94.4	91.7	100.0

The type strains for each species: 1. CCUG 56438^T; 2. CCUG 57103^T; 3. CCUG 724^T; 4. CCUG 407^T; 5. CCUG 47057^T; 6. CCUG 47062^T; 7. CCUG 56371^T; 8. CCUG 61966^T; 9. CCUG 61961^T; 10. CCUG 61972^T; 11. CCUG 61968^T.

(b)

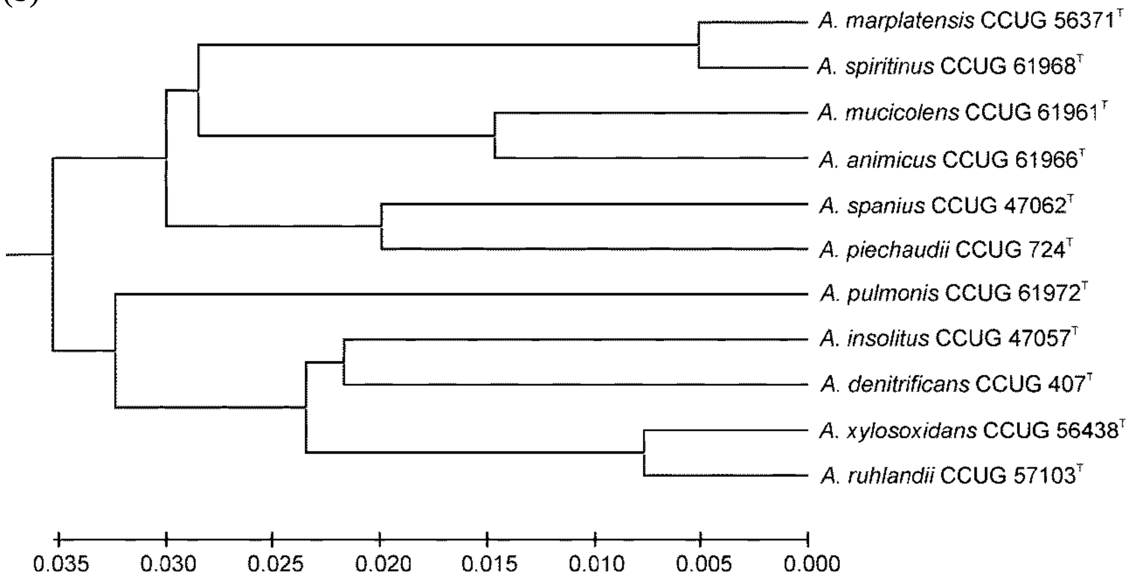

Supplemental Figure S4. Gene sequence similarities and evolutionary relationships for the type strains of the *Achromobacter* species. (a) *recA* gene sequence similarities for the type strains of the *Achromobacter* species gene sequence similarities are on the lower diagonal; aminoacid sequence similarities are on the upper diagonal. (b) The sequence relationships were inferred, using the UPGMA method. The analysis involved 11 nucleotide sequences. All positions containing gaps and missing data were eliminated. There were a total of 621 positions in the final dataset.

(a)

	1	2	3	4	5	6	7	8	9	10	11
1. *A. xylosoxidans*	100.0	99.3	97.9	99.3	99.6	97.9	97.9	99.6	99.3	97.9	99.0
2. *A. ruhlandii*	97.6	100.0	98.3	100.0	99.7	98.3	98.3	99.7	100.0	98.3	99.7
3. *A. piechaudii*	91.3	91.3	100.0	98.3	98.3	100.0	100.0	98.3	98.3	100.0	98.0
4. *A. denitrificans*	94.9	94.9	90.7	100.0	99.7	98.3	98.3	99.7	100.0	98.3	99.7
5. *A. insolitus*	93.6	93.1	90.7	93.6	100.0	98.3	98.3	100.0	99.7	98.3	99.4
6. *A. spanius*	90.3	91.5	94.5	91.0	90.7	100.0	100.0	98.3	98.3	100.0	98.0
7. *A. marplatensis*	92.4	92.6	93.7	92.3	91.5	93.7	100.0	98.3	98.3	100.0	98.0
8. *A. animicus*	93.9	94.9	91.0	93.6	93.4	91.6	92.9	100.0	99.7	98.3	99.4
9. *A. mucicolens*	93.6	94.5	90.5	93.4	92.6	90.3	92.1	96.0	100.0	98.3	99.7
10. *A. pulmonis*	95.8	96.5	89.9	93.9	92.0	89.9	92.0	93.2	93.2	100.0	98.0
11. *A. spiritinus*	92.3	92.4	93.7	92.1	91.2	93.2	97.9	92.9	92.0	91.3	100.0

The type strains for each species: 1. CCUG 56438^{T}; 2. CCUG 57103^{T}; 3. CCUG 724^{T}; 4. CCUG 407^{T}; 5. CCUG 47057^{T}; 6. CCUG 47062^{T}; 7. CCUG 56371^{T}; 8. CCUG 61966^{T}; 9. CCUG 61961^{T}; 10. CCUG 61972^{T}; 11. CCUG 61968^{T}.

(b)

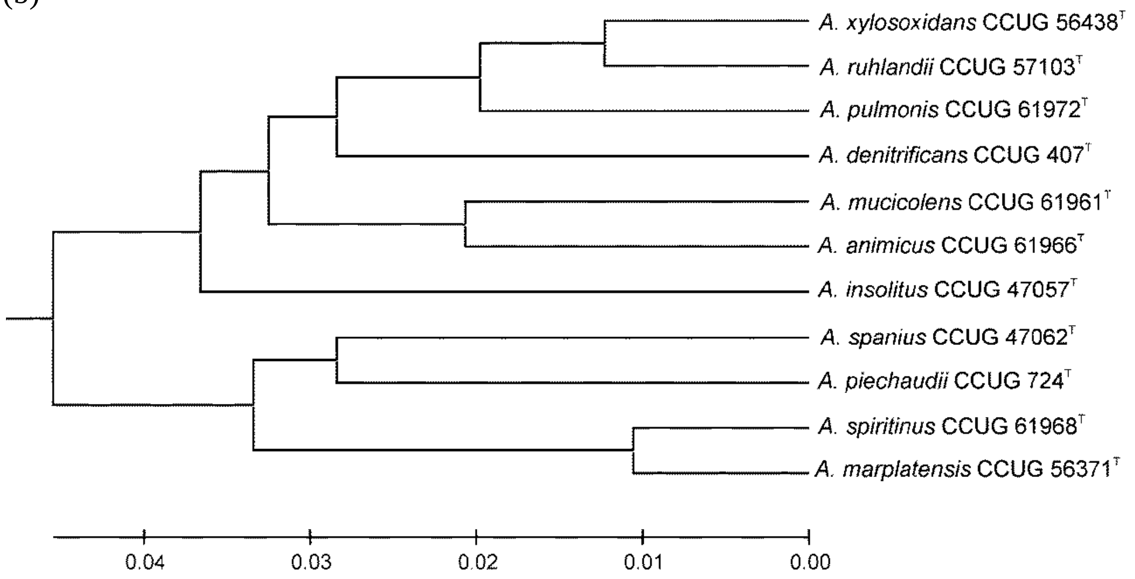

Supplemental Figure S5. Gene sequence similarities and evolutionary relationships for the type strains of the *Achromobacter* species. (a) *rpoB* gene sequence similarities for the type strains of the *Achromobacter* species gene sequence similarities are on the lower diagonal; aminoacid sequence similarities are on the upper diagonal. (b) The sequence relationships were inferred, using the UPGMA method. The analysis involved 11 nucleotide sequences. All positions containing gaps and missing data were eliminated. There were a total of 598 positions in the final dataset.

(a)

	1	2	3	4	5	6	7	8	9	10	11
1. *A. xylosoxidans*	100.0	99.7	98.5	99.2	98.9	98.5	98.5	99.7	99.4	99.7	98.5
2. *A. ruhlandii*	98.7	100.0	98.2	99.4	99.2	98.2	98.2	99.4	99.2	100.0	98.2
3. *A. piechaudii*	93.0	92.3	100.0	98.4	98.2	100.0	100.0	98.4	98.7	98.2	100.0
4. *A. denitrificans*	96.2	96.2	93.3	100.0	99.7	98.4	98.4	99.4	99.7	99.4	98.4
5. *A. insolitus*	94.8	94.8	93.3	97.3	100.0	98.2	98.2	99.2	99.4	99.2	98.2
6. *A. spanius*	92.8	92.1	96.5	92.8	92.7	100.0	100.0	98.4	98.7	98.2	100.0
7. *A. marplatensis*	94.3	93.7	96.3	94.0	93.8	96.7	100.0	98.4	98.7	98.2	100.0
8. *A. animicus*	95.0	94.5	93.5	95.5	94.8	92.7	94.0	100.0	99.7	99.4	98.4
9. *A. mucicolens*	94.7	94.5	94.0	96.2	95.2	92.8	94.3	98.3	100.0	99.2	98.7
10. *A. pulmonis*	98.0	99.0	92.3	95.8	94.8	92.1	93.3	94.2	94.2	100.0	98.2
11. *A. spiritinus*	94.3	93.7	96.2	94.0	94.0	96.5	99.8	93.8	94.2	93.3	100.0

The type strains for each species: 1. CCUG 56438^T; 2. CCUG 57103^T; 3. CCUG 724^T; 4. CCUG 407^T; 5. CCUG 47057^T; 6. CCUG 47062^T; 7. CCUG 56371^T; 8. CCUG 61966^T; 9. CCUG 61961^T; 10. CCUG 61972^T; 11. CCUG 61968^T.

(b)

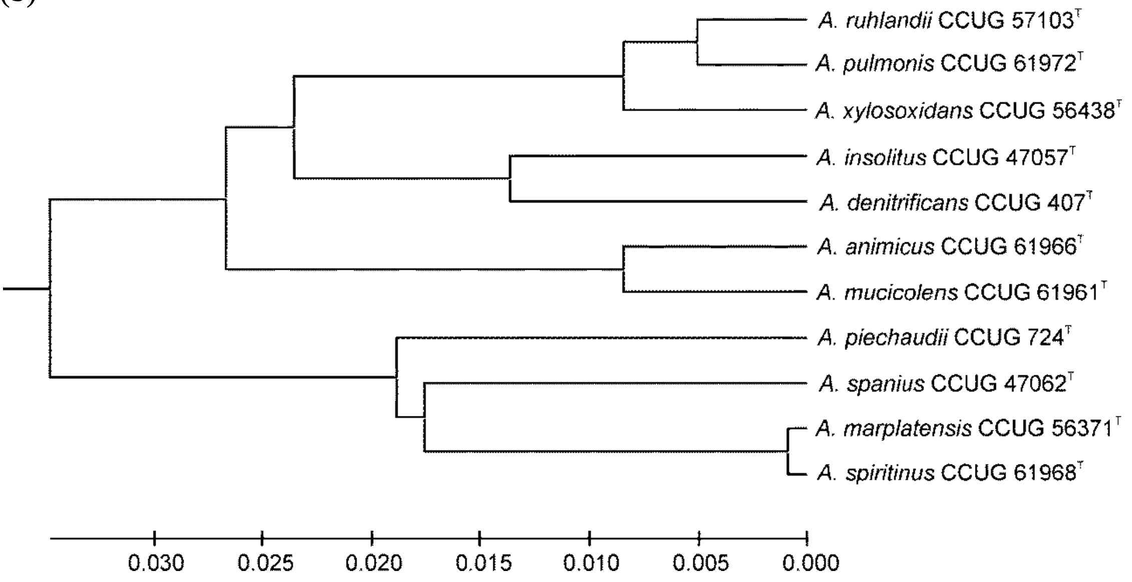

Supplemental Figure S6. Phylogenetic tree of the 57 *Achromobacter* strains studied based on the analysis of 513 bp of the *atpD* gene. Distance matrix was calculated by the Jukes-Cantor method. Dendrogram was generated by neighbor-joining. *Bordetella pertussis* CCUG 30873^{T} was used as an outgroup. The bar indicates sequence divergence. Bootstrap values of more than 500 (from 1000 replicates) are indicated at the nodes.

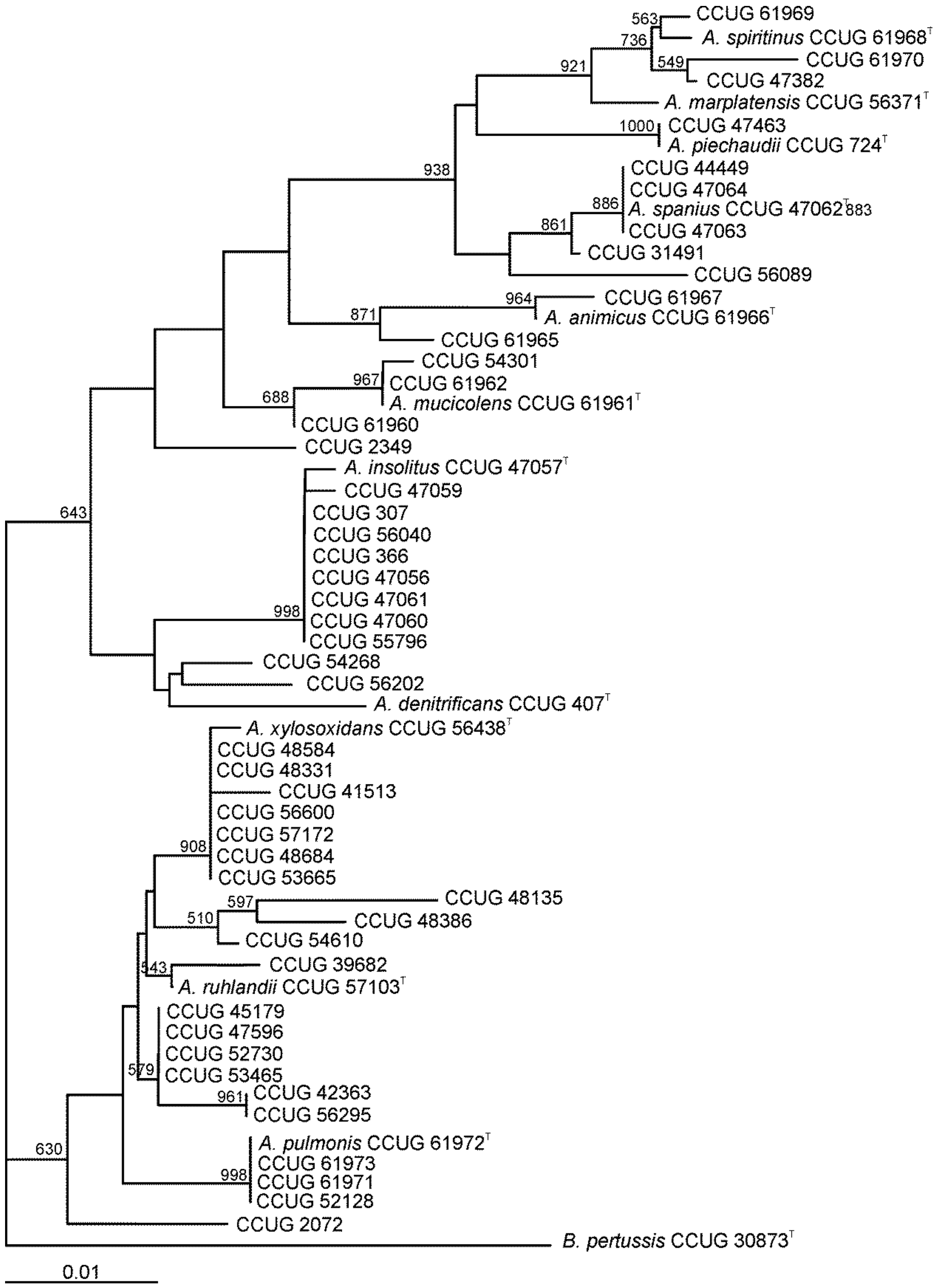

Supplemental Figure S7. Phylogenetic tree of the 57 *Achromobacter* strains studied based on the analysis of 528 bp of the *gyrB* gene. Distance matrix was calculated by the Jukes-Cantor method. Dendrogram was generated by neighbor-joining. *Bordetella pertussis* CCUG 30873^T was used as an outgroup. The bar indicates sequence divergence. Bootstrap values of more than 500 (from 1000 replicates) are indicated at the nodes.

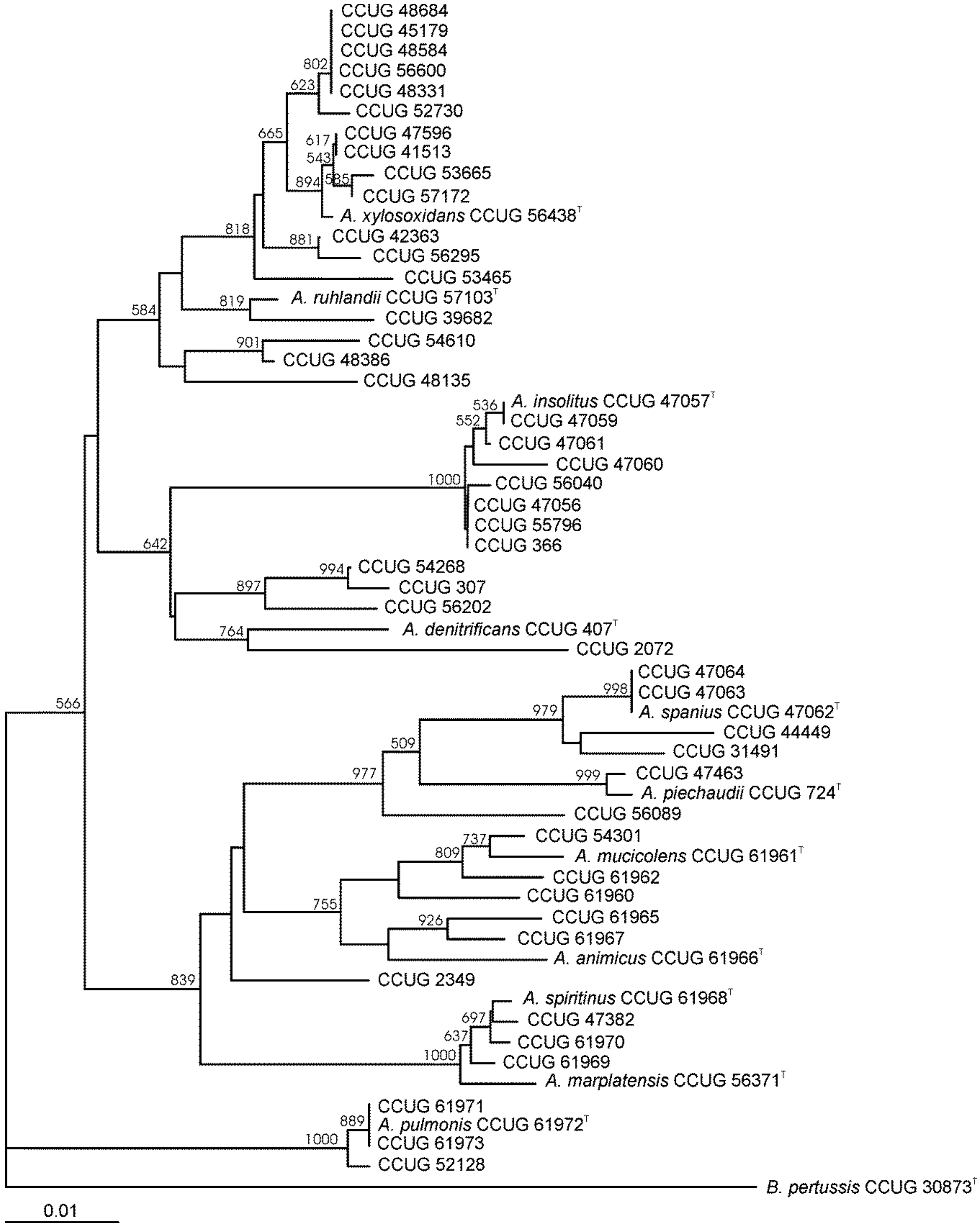

Supplementary Figure S8. Phylogenetic tree of the 57 *Achromobacter* strains studied based on the analysis of 527 bp of the *rpoB* gene. Distance matrix was calculated by the Jukes-Cantor method. Dendrogram was generated by neighbor-joining. *Bordetella pertussis* CCUG 30873^T was used as an outgroup. The bar indicates sequence divergence. Bootstrap values of more than 500 (from 1000 replicates) are indicated at the nodes.

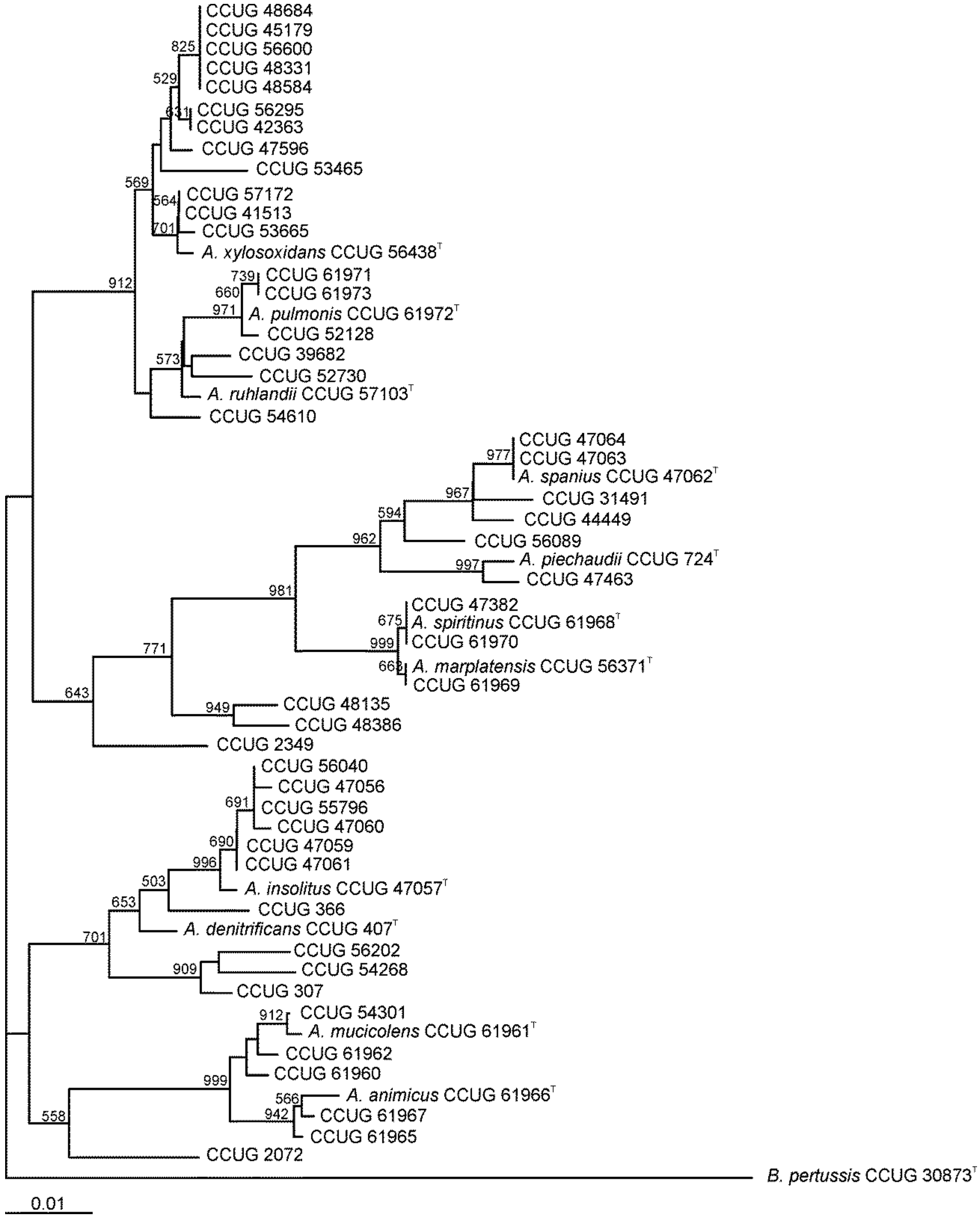

Supplemental Figure S9. Phylogenetic tree of the 57 *Achromobacter* strains studied based on the analysis of 398 bp of the 16S rRNA gene. Distance matrix was calculated by the Jukes-Cantor method. Dendrogram was generated by neighbor-joining. *Bordetella pertussis* CCUG 30873^T was used as an outgroup. The bar indicates sequence divergence. Bootstrap values of more than 500 (from 1000 replicates) are indicated at the nodes.

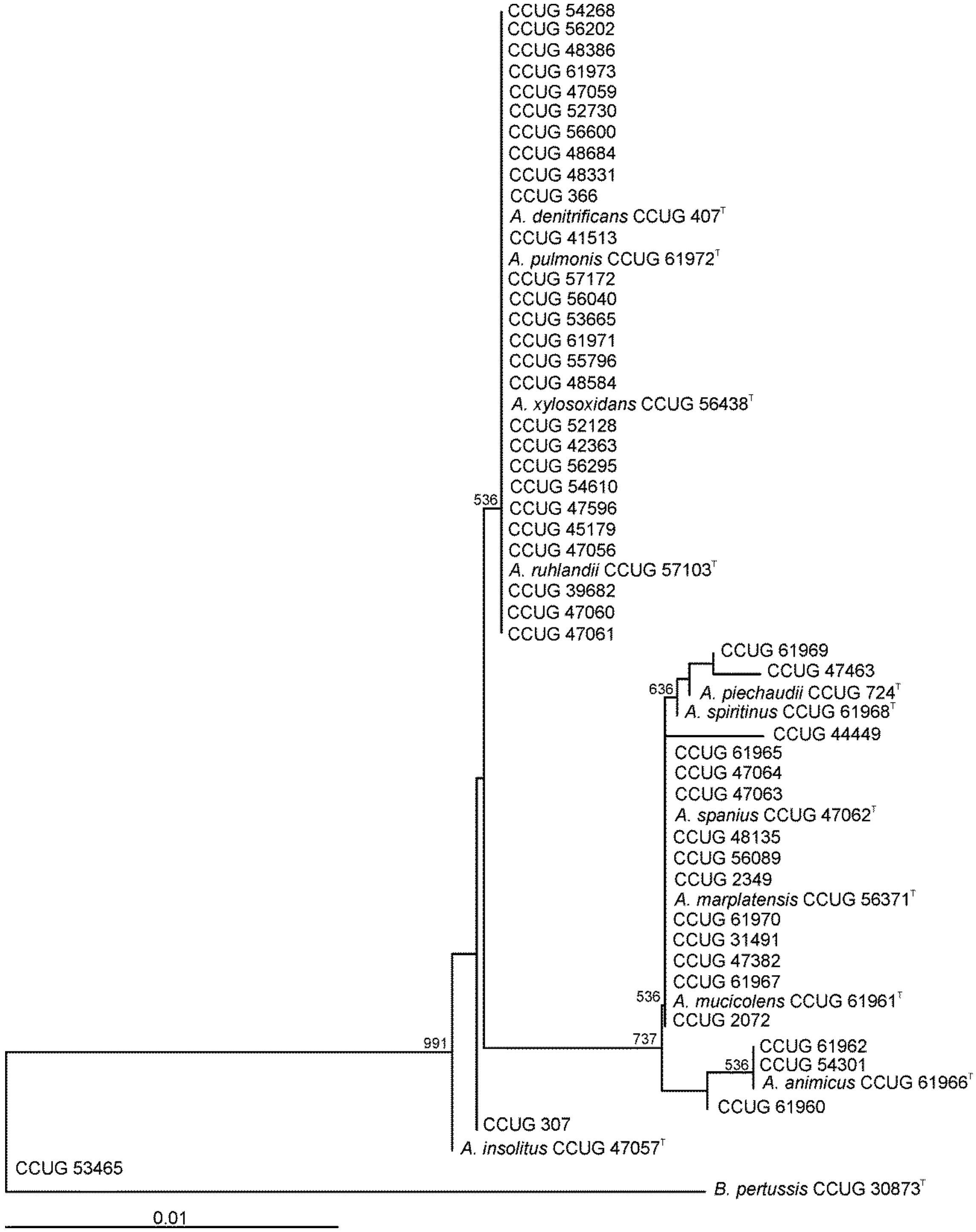

Supplementary Figure S10. Phylogenetic tree of the strains of *Achromobacter* used in this study based on the phylogenetic analysis of four concatenated genes (*atpD, gyrB, recA* and *rpoB*). Distance matrices were calculated by the Jukes-Cantor method. Dendrograms were generated by neighbor-joining. *Bordetella pertussis* CCUG 30873^T was used as an outgroup. The bar indicates sequence divergence. Bootstrap values of more than 500 (from 1000 replicates) are indicated at the nodes.

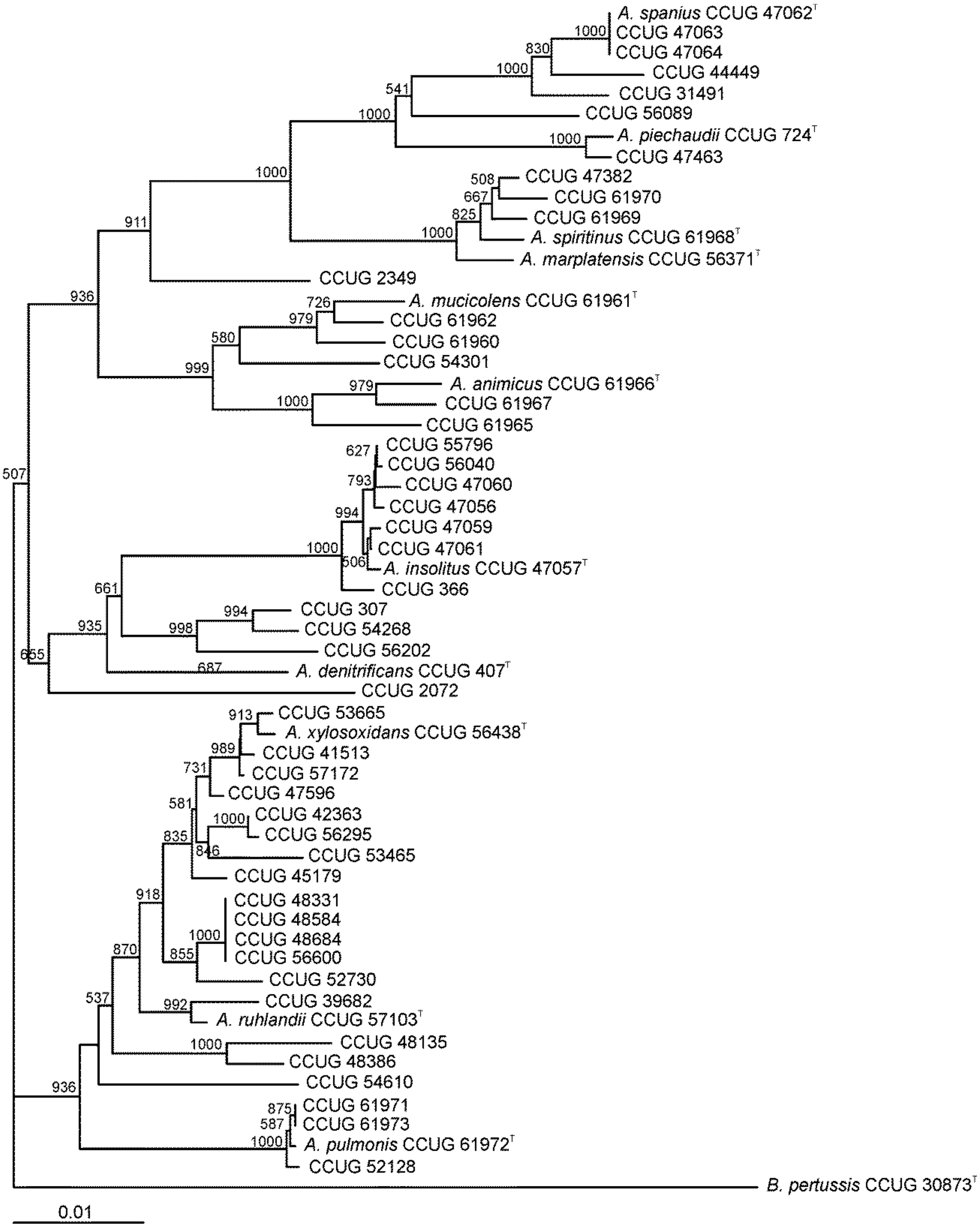

Supplemental Figure S11. Gene sequence similarities and evolutionary relationships for the type strains of the *Achromobacter* species. (a) *nrdA* gene sequence similarities of a 765 pb region for the type strains of the *Achromobacter* species gene sequence similarities are on the lower diagonal; *nrdA* gene sequence similarities of the short region are on the upper diagonal.

(a)

	1	2	3	4	5	6	7	8	9	10	11
1. *A. xylosoxidans*	100.0	98.7	93.3	94.4	93.1	93.5	93.1	94.0	93.5	97.1	93.1
2. *A. ruhlandii*	98.8	100.0	93.8	94.2	94.0	94.0	92.7	94.0	94.0	97.6	92.7
3. *A. piechaudii*	92.2	92.7	100.0	92.4	92.9	97.1	92.7	94.4	94.0	93.3	92.7
4. *A. denitrificans*	93.3	93.5	90.5	100.0	95.3	92.9	92.0	94.0	93.5	94.0	92.4
5. *A. insolitus*	93.6	94.4	92.4	94.0	100.0	94.0	91.8	94.0	93.3	94.0	92.2
6. *A. spanius*	92.7	93.2	96.2	91.1	93.6	100.0	95.1	94.4	95.5	93.1	95.1
7. *A. marplatensis*	92.8	92.8	93.9	92.0	92.9	95.2	100.0	93.8	94.0	91.8	98.9
8. *A. animicus*	94.1	94.2	93.2	93.5	94.6	93.6	94.4	100.0	98.2	93.1	93.8
9. *A. mucicolens*	93.9	94.4	93.5	93.1	94.5	94.8	94.8	98.2	100.0	93.1	94.0
10. *A. pulmonis*	96.9	97.3	91.9	93.3	94.0	92.2	91.6	93.5	93.2	100.0	92.2
11. *A. spiritinus*	92.8	92.8	93.9	92.3	93.2	95.2	99.3	94.4	94.8	91.9	100.0

The type strains for each species: 1. CCUG 56438^{T}; 2. CCUG 57103^{T}; 3. CCUG 724^{T}; 4. CCUG 407^{T}; 5. CCUG 47057^{T}; 6. CCUG 47062^{T}; 7. CCUG 56371^{T}; 8. CCUG 61966^{T}; 9. CCUG 61961^{T}; 10. CCUG 61972^{T}; 11. CCUG 61968^{T}. nrdA and nrdA-765 gene sequences have been taken from PubMLST *Achromobacter* web page.
